# Supplementary material for: Purging of highly deleterious mutations through severe bottlenecks in Alpine ibex
Source: Nat Commun. 2020 Feb 21;11:1001. doi: 10.1038/s41467-020-14803-1 (PMC7035315; doi:10.1038/s41467-020-14803-1)
Supplement: Supplementary file 1 — Supplementary Information [file 41467_2020_14803_MOESM1_ESM.pdf]

Supplementary information

Purging of highly deleterious mutations through severe bottlenecks in ibex

Grossen et al.

TABLE OF CONTENTS

Supplementary Figure 1 3
Supplementary Figure 2A 3
Supplementary Figure 2B 3
Supplementary Figure 3A 4
Supplementary Figure 3B 5
Supplementary Figures 4A - 4D 6
Supplementary Figures 5A - 5D 7
Supplementary Figure 6 8
Supplementary Figure 7A 9
Supplementary Figure 7B 10
Supplementary Figure 7C 10
Supplementary Figure 7D 11
Supplementary Figure 8 12
Supplementary Figure 9 12
Supplementary Figure 10 13
Supplementary Figure 13A 16
Supplementary Figure 13B 16
Supplementary Figure 14 17
Supplementary Figure 15 18
Supplementary Figure 18 21
Supplementary Figure 19 22
Supplementary Figure 20 23
Supplementary Figure 21 24
Supplementary Figure 22: 25
Supplementary Figures 23 – 31 26
Supplementary Figures 32 - 39 31
Supplementary Figure 45 39
Supplementary Table 1A 40
Supplementary Table 1B 41
Supplementary Table 2 42
Supplementary Table 3 43
Supplementary Table 4 45
Supplementary Table 5 45
Supplementary Table 6 46
Supplementary Note 1 48
Supplementary References 50

SUPPLEMENTARY FIGURES

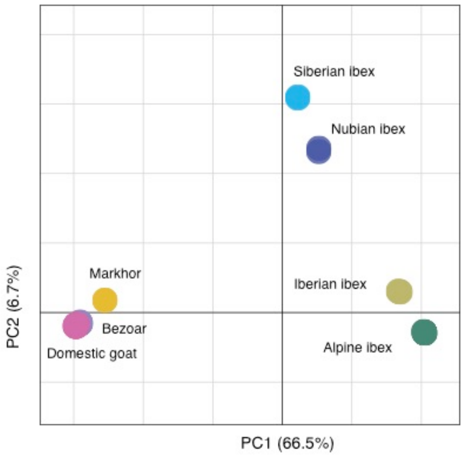

Supplementary Figure 1: Principal component analysis. All ibex species under study are included. Colors indicate different species.

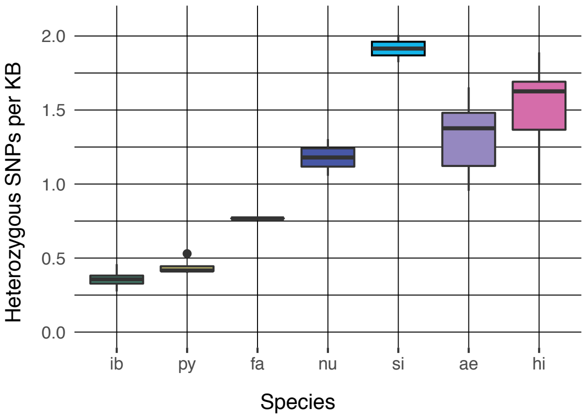

Supplementary Figure 2A: Genome-wide heterozygosity per species. Heterozygosity is expressed as the number of heterozygous autosomal SNPs per kb. ib: *Capra ibex*, py: *C. pyrenaica*, fa: *C. falconeri*, nu: *C. nubiana*, si: *C. sibirica*, ae: *C. aegagrus*, hi: *C. hircus*. Box plots show the median, the 25th and 75th percentiles, Tukey whiskers (median ± 1.5 times interquartile range), and outliers (•).

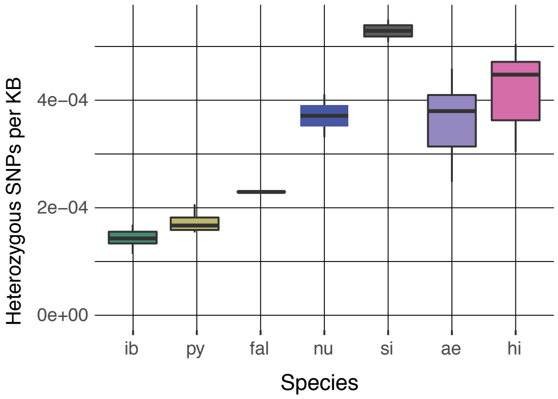

Supplementary Figure 2B: Genome-wide heterozygosity per species in coding sequences (CDS). Heterozygosity is expressed as the number of heterozygous SNPs per kb of CDS. ib: *Capra ibex*, py: *C. pyrenaica*, fa: *C. falconeri*, nu: *C. nubiana*, si: *C. sibirica*, ae: *C. aegagrus*, hi: *C. hircus*. Box plot elements as in Supplementary Figure 2A.

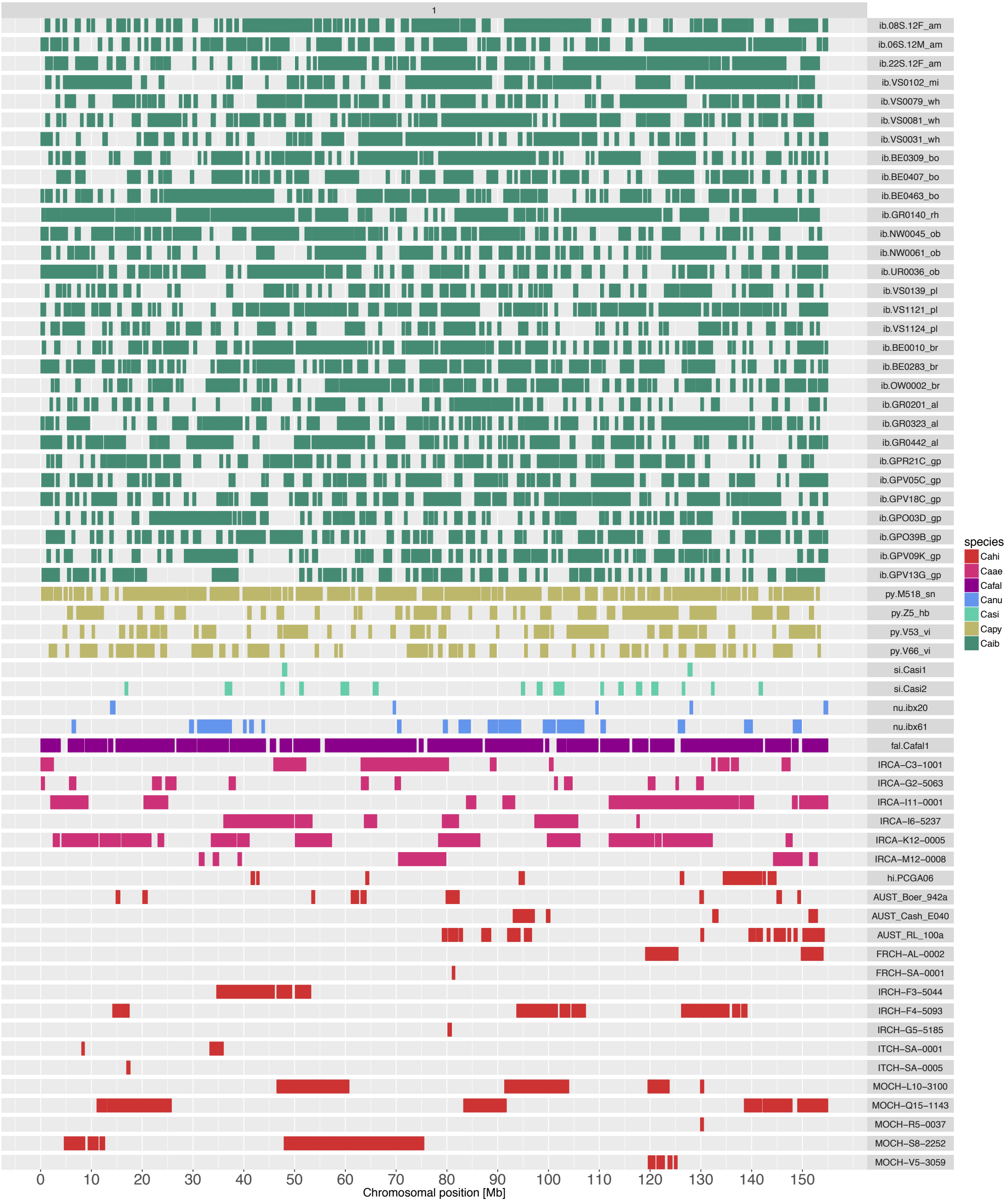

Supplementary Figure 3A: Runs of homozygosity (ROH) along chromosome 1. Bcftools analysis with viterbi-training option turned off. The alternative allele frequency prior was set to 0.4. Colors represent species as in Supplementary Figures 1 and 2. Caib: *Capra ibex*, Capy: *C. pyrenaica*, Cafal: *C. falconeri*, Canu: *C. nubiana*, Casi: *C. sibirica*, Caae: *C. aegagrus*, Cahi: *C. hircus*.

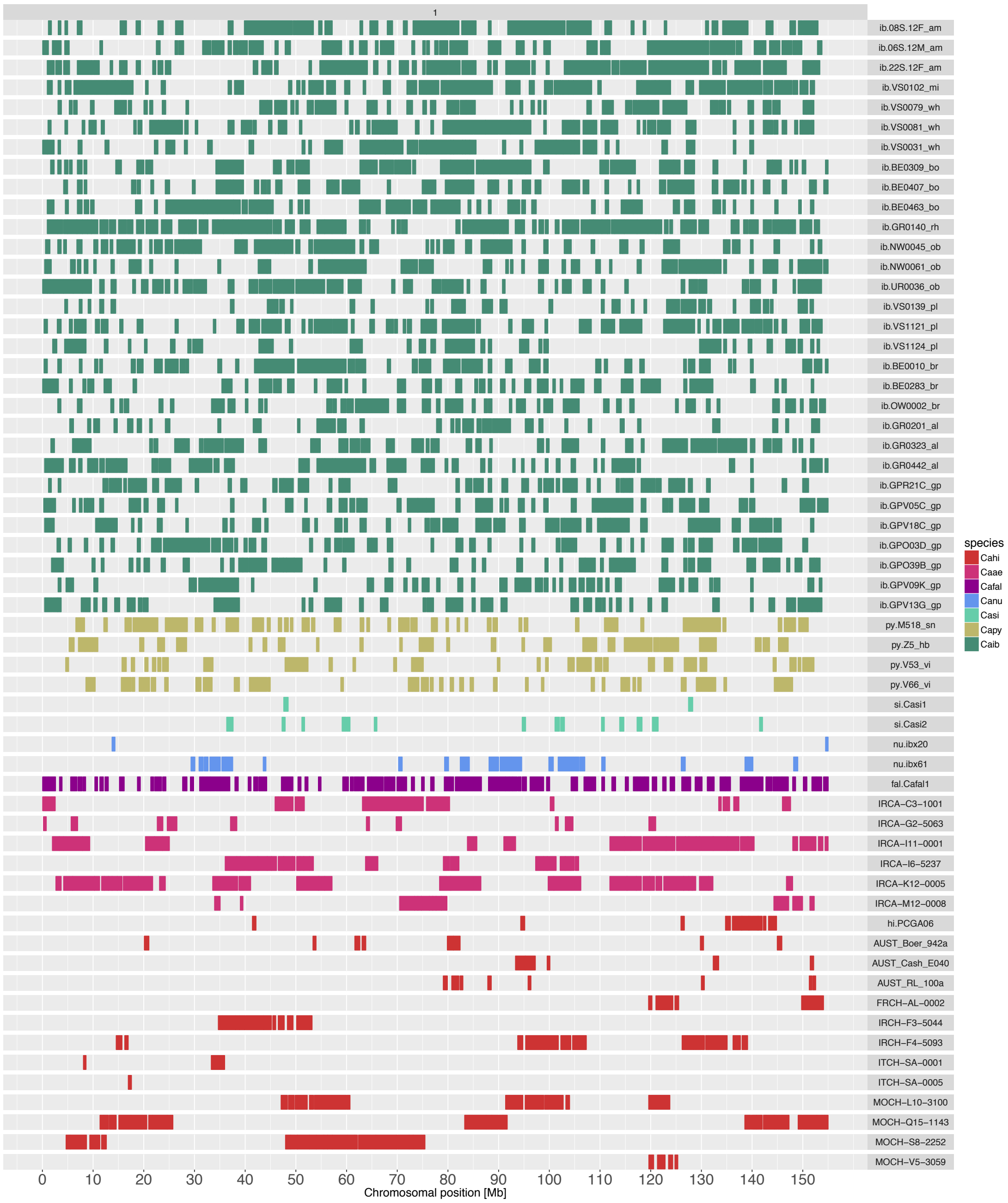

Supplementary Figure 3B: Runs of homozygosity (ROH) along chromosome 1. Bcftools was run with the viterbi-training option turned on. The alternative allele frequency prior was set to 0.4. Colors represent species as in Supplementary Figures 1 and 2. Caib: *Capra ibex*, Capy: *C. pyrenaica*, Cafal: *C. falconeri*, Canu: *C. nubiana*, Casi: *C. sibirica*, Caae: *C. aegagrus*, Cahi: *C. hircus*.

Supplementary Figures 4A - 4D: Runs of homozygosity (ROH) analysis with different bcftools settings. Red lines show estimated state (0 corresponds to ROH region, 1 to non-ROH region). The blue line shows heterozygosity in a sliding window (window size=100 bp, step size=1 bp) along chromosomal positions. Data is shown for the *C. ibex* individual GPR21C. The window represents chromosome 23 and positions 15-30 Mb.

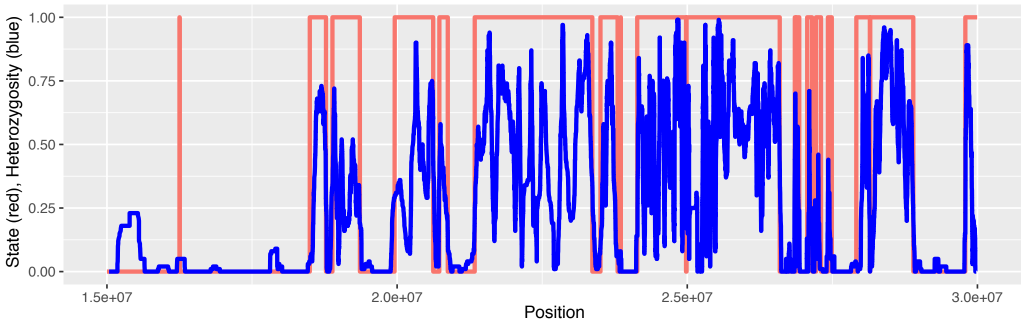

Supplementary Figure 4A: Allele frequency prior (af.dtlt) set to 0.4.

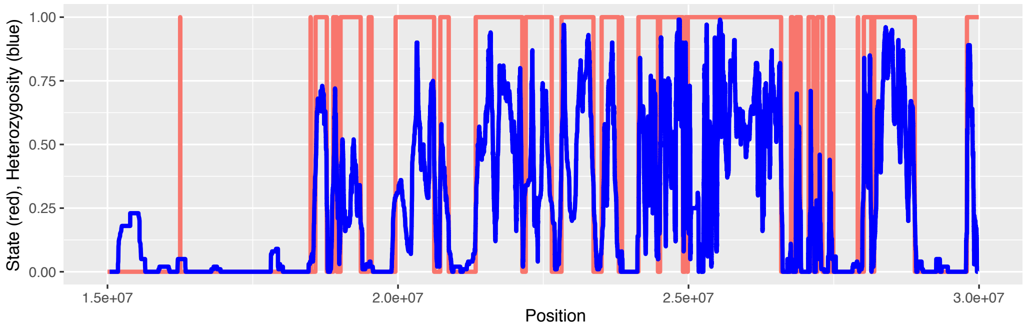

Supplementary Figure 4B: Allele frequency prior (af.dtlt) set to 0.4 and viterbi-training option turned on.

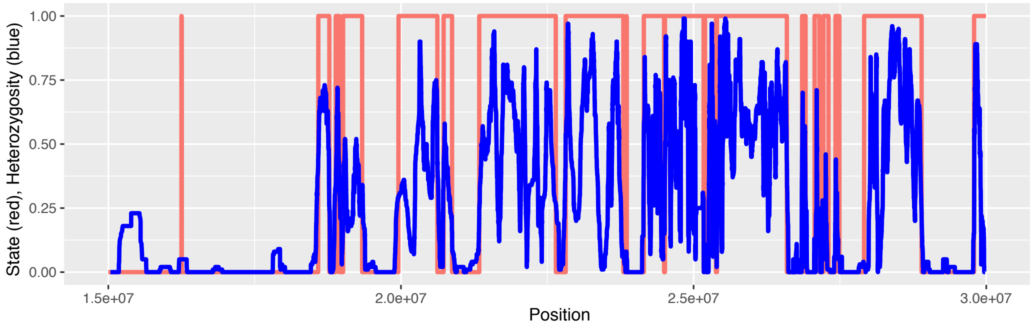

Supplementary Figure 4C: Allele frequency priors were set to allele frequencies observed in the Gran Paradiso population.

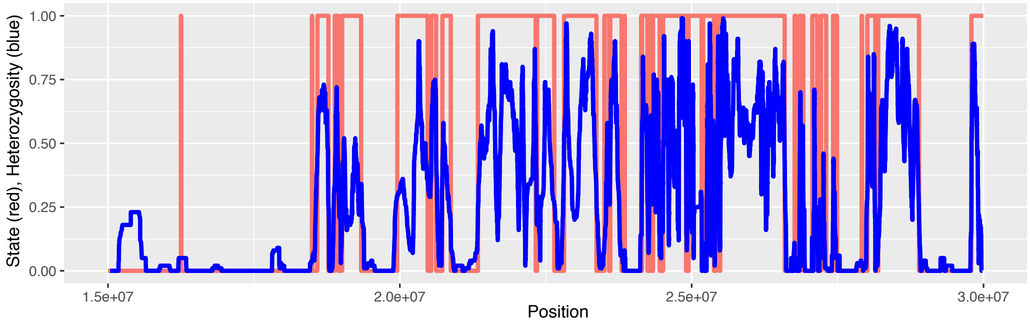

Supplementary Figure 4D: Allele frequency priors were set to allele frequencies observed in the Gran Paradiso population and viterbi-training option turned on.

Supplementary Figures 5A - 5D: Runs of homozygosity (ROH) analysis with different bcftools settings. Red lines show estimated state (0 corresponds to ROH region, 1 to non-ROH region). The blue line shows heterozygosity in a sliding window (window size=100 bp, step size=1 bp) along chromosomal positions. Data is shown for the *C. ibex* individual GPV05C. The window represents chromosome 23 and positions 15-30 Mb.

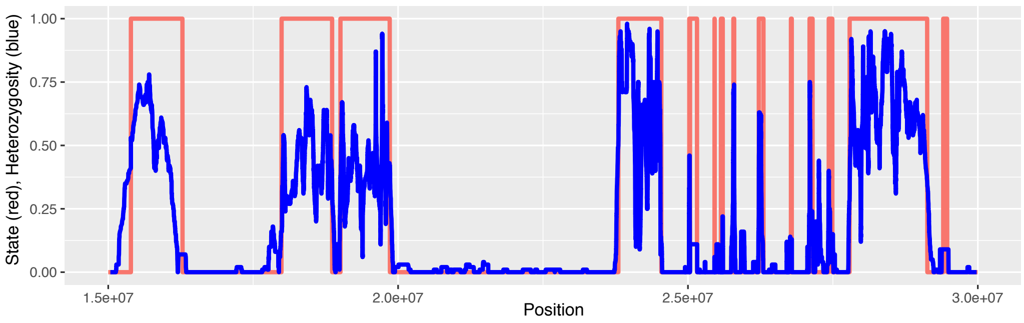

Supplementary Figure 5A: Allele frequency prior (af.dtlt) set to 0.4.

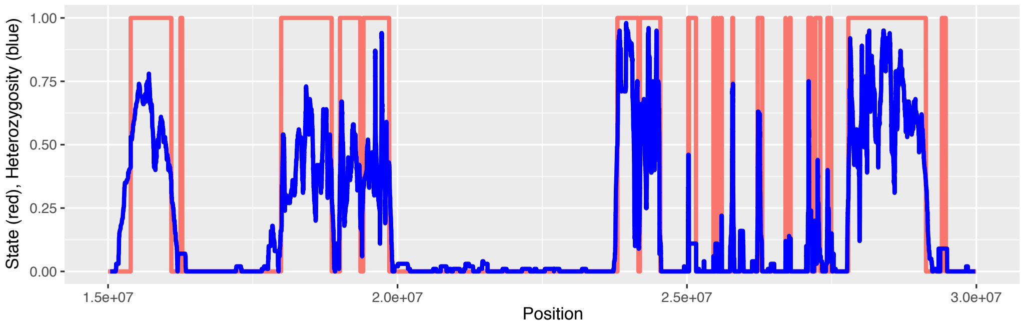

Supplementary Figure 5B: Allele frequency prior (af.dtlt) set to 0.4 and viterbi-training option turned on.

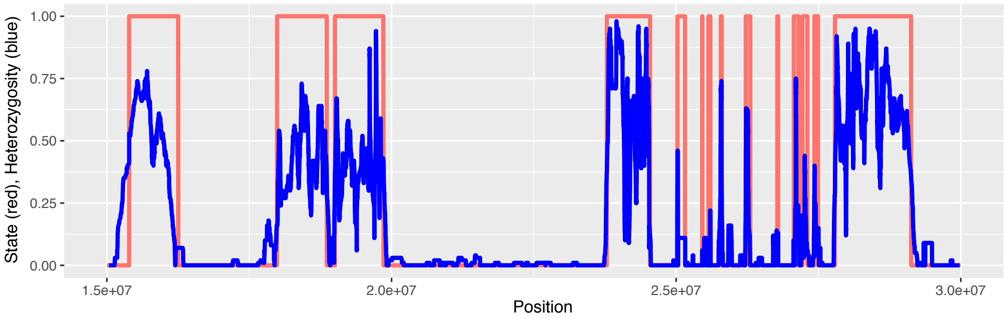

Supplementary Figure 5C: Allele frequency priors were set to allele frequencies observed in the Gran Paradiso population.

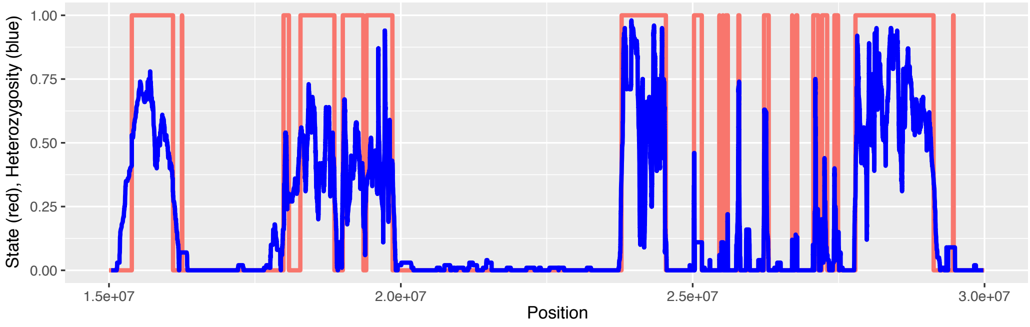

Supplementary Figure 5D: Allele frequency priors were set to allele frequencies observed in the Gran Paradiso population and viterbi-training option turned on.

Alignment of trimmed reads to domestic goat reference genome (CHIR_1.0) and mark duplicates (bowtie2)

↓

SNP calling GATK (HaplotypeCaller, GenotypeGVCF, VariantFiltration) and Freebayes

↓

SNP annotation (snpEff)

↓

Only retain high quality mutations in conserved regions:
GERP > -2
FPKM > 0.3 in at least one of 10 organs

↓

Categorization:
snpEff predicted impact: High, Moderate, Low, Modifier
GERP scores: > 6, 4 to 6, 2 to 4, -2 to 2

Supplementary Figure 6: Pipeline for SNP calling, validation and categorization of mutational impact. See Methods for details on each step.

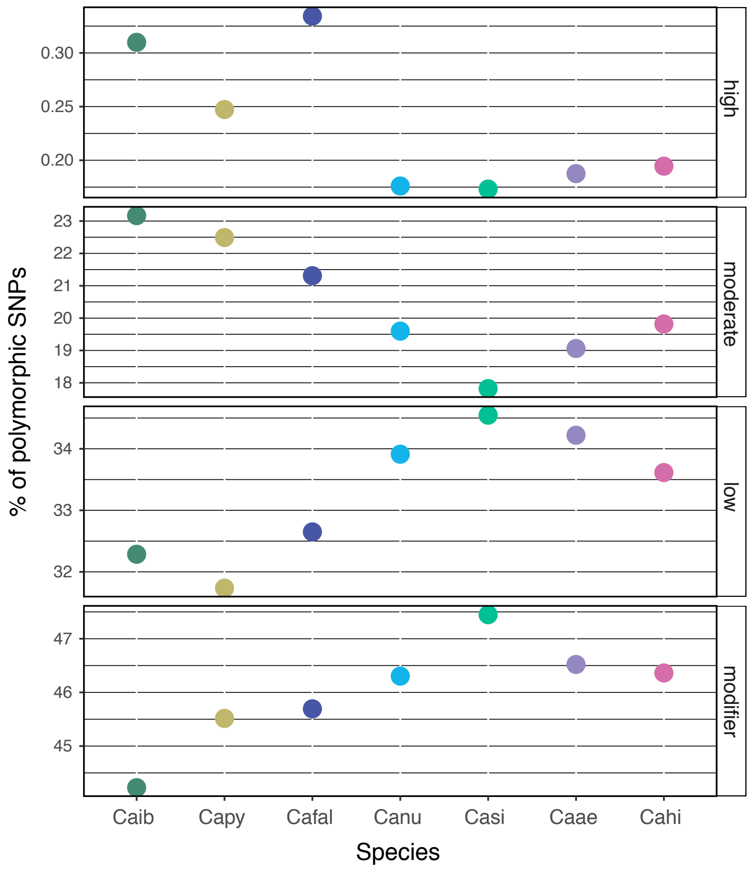

Supplementary Figure 7A: Deleterious mutations in ibex species (SnpEff analyses). Proportion of single nucleotide polymorphisms (SNPs) polymorphic within species categorized into four impact categories according to SnpEff (high, moderate, low, modifier). Caib: *Capra ibex*, Capy: *C. pyrenaica*, Cafal: *C. falconeri*, Canu: *C. nubiana*, Casi: *C. sibirica*, Caae: *C. aegagrus*, Cahi: *C. hircus*.

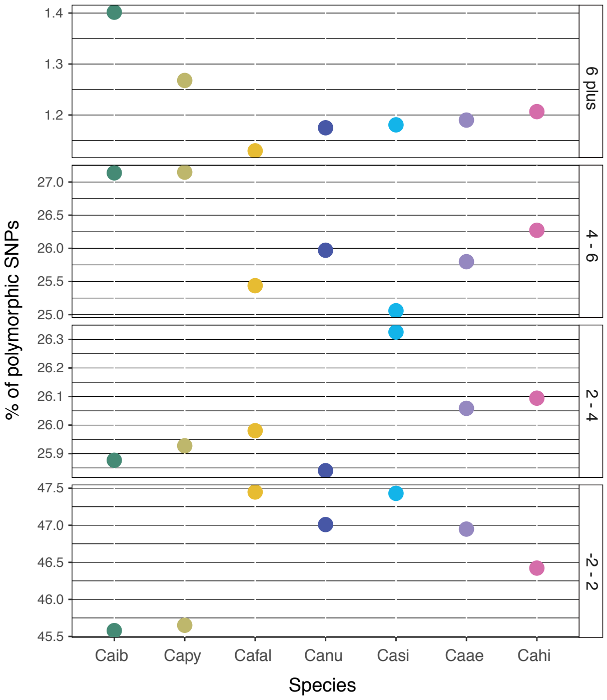

Supplementary Figure 7B: Deleterious mutations in ibex species (GERP score analyses). Proportion of single nucleotide polymorphisms (SNPs) polymorphic within species categorized into GERP score ranges (>6, 4 - 6, 2 - 4, -2 - 2). Caib: *Capra ibex*, Capy: *C. pyrenaica*, Cafal: *C. falconeri*, Canu: *C. nubiana*, Casi: *C. sibirica*, Caae: *C. aegagrus*, Cahi: *C. hircus*.

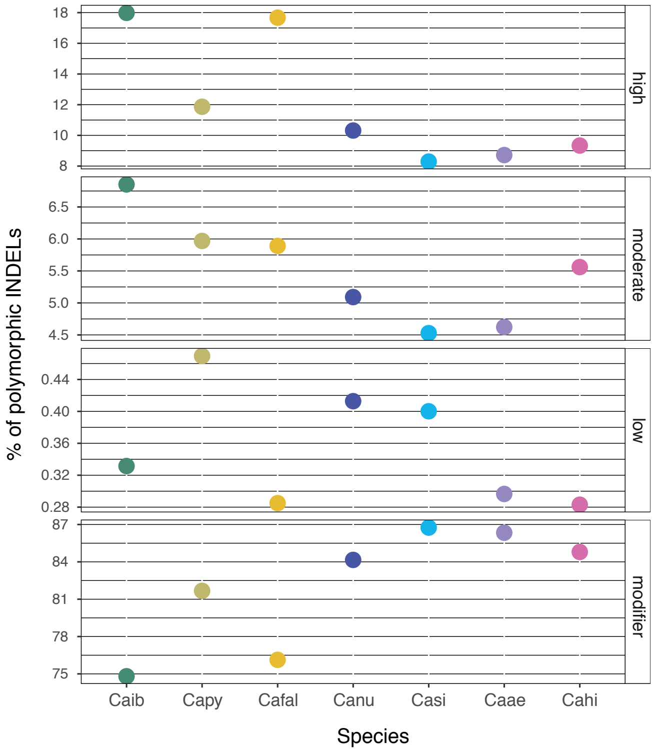

Supplementary Figure 7C: Deleterious mutations in ibex species (indel analyses based on SnpEff). Proportion of indels (≤10 bp) polymorphic within species categorized into four impact categories according to SnpEff (high, moderate, low, modifier). Caib: *Capra ibex*, Capy: *C. pyrenaica*, Cafal: *C. falconeri*, Canu: *C. nubiana*, Casi: *C. sibirica*, Caae: *C. aegagrus*, Cahi: *C. hircus*.

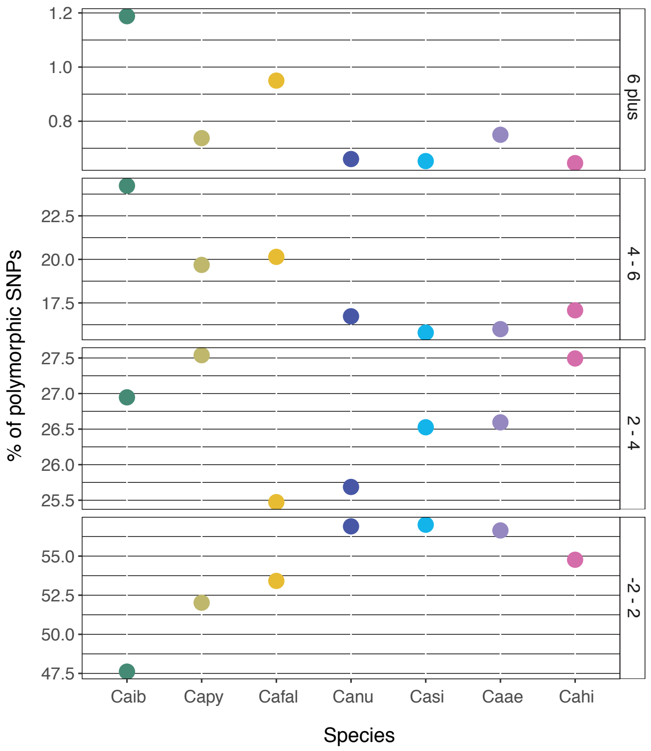

Supplementary Figure 7D: Deleterious mutations in ibex species (indel analyses based on GERP scores). Proportion of variant indels (up to 10 bp long) polymorphic within species categorized into GERP score ranges (>6, 4 - 6, 2 - 4, -2 - 2). Caib: *Capra ibex*, Capy: *C. pyrenaica*, Cafal: *C. falconeri*, Canu: *C. nubiana*, Casi: *C. sibirica*, Caae: *C. aegagrus*, Cahi: *C. hircus*.

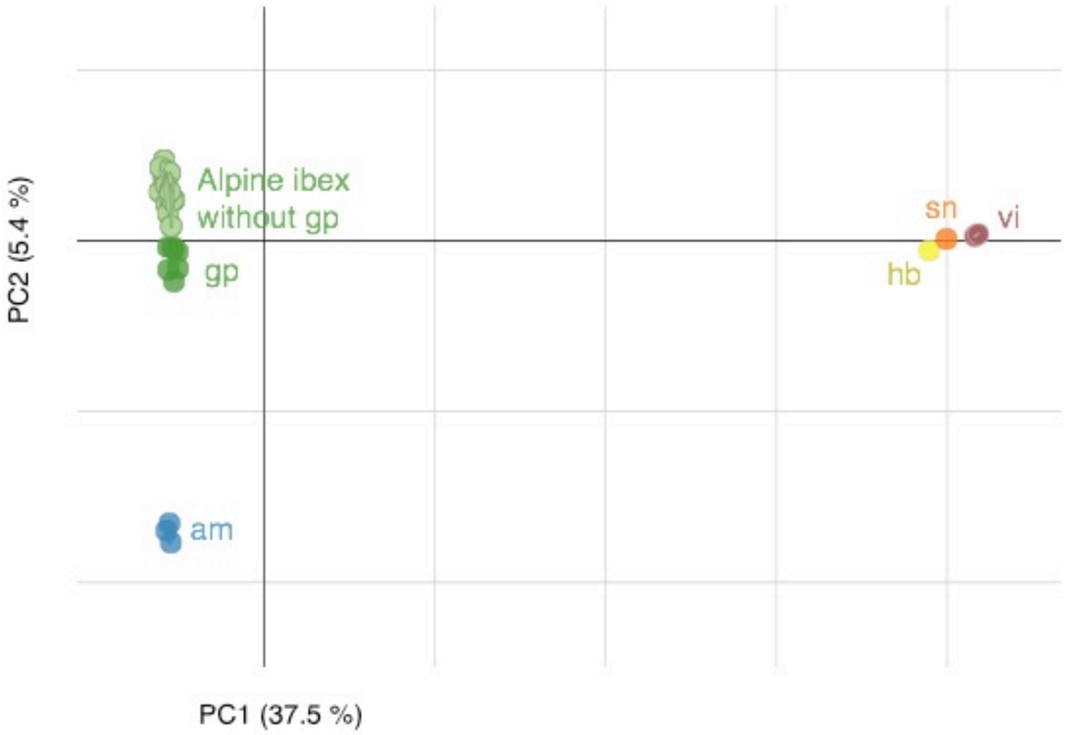

Supplementary Figure 8: Principal component analyses of Alpine and Iberian ibex. Alpine ibex: Dark green: Gran Paradiso {gp}, light green: Swiss individuals, blue: Alpi Marittime {am}. Iberian ibex: yellow: Maestrazgo {hb}, orange: Sierra Nevada {sn}, brown: Victoriae {vi}.

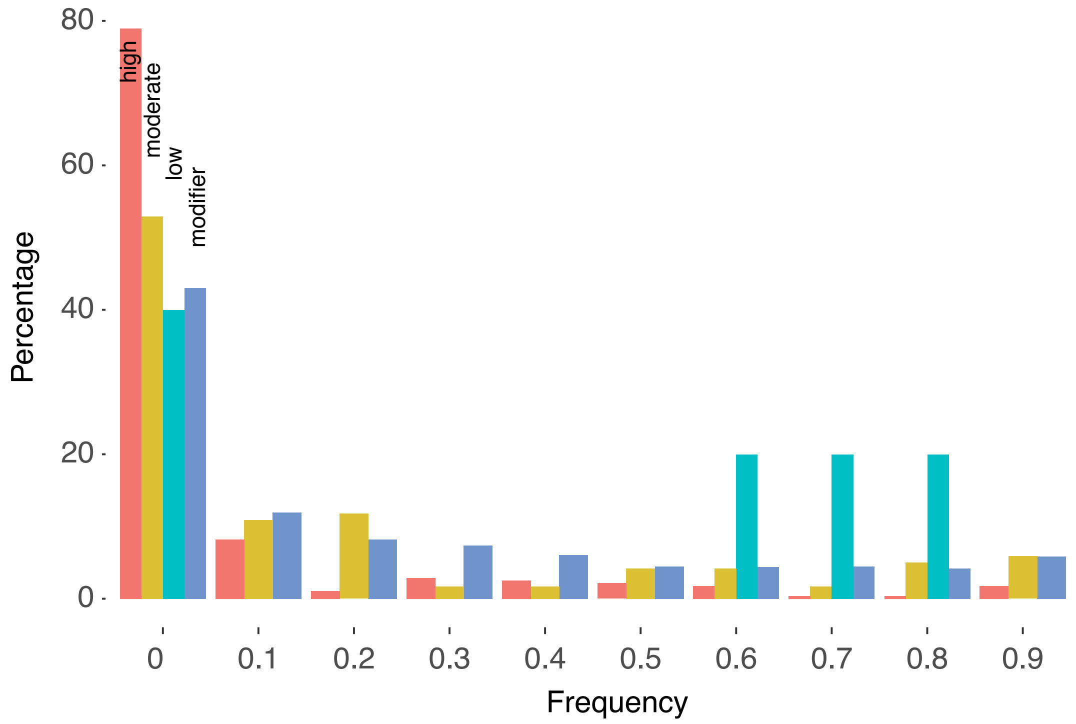

Supplementary Figure 9: Site-frequency spectrum (SFS) of different SnpEff categories. Indel mutations in Alpine ibex.

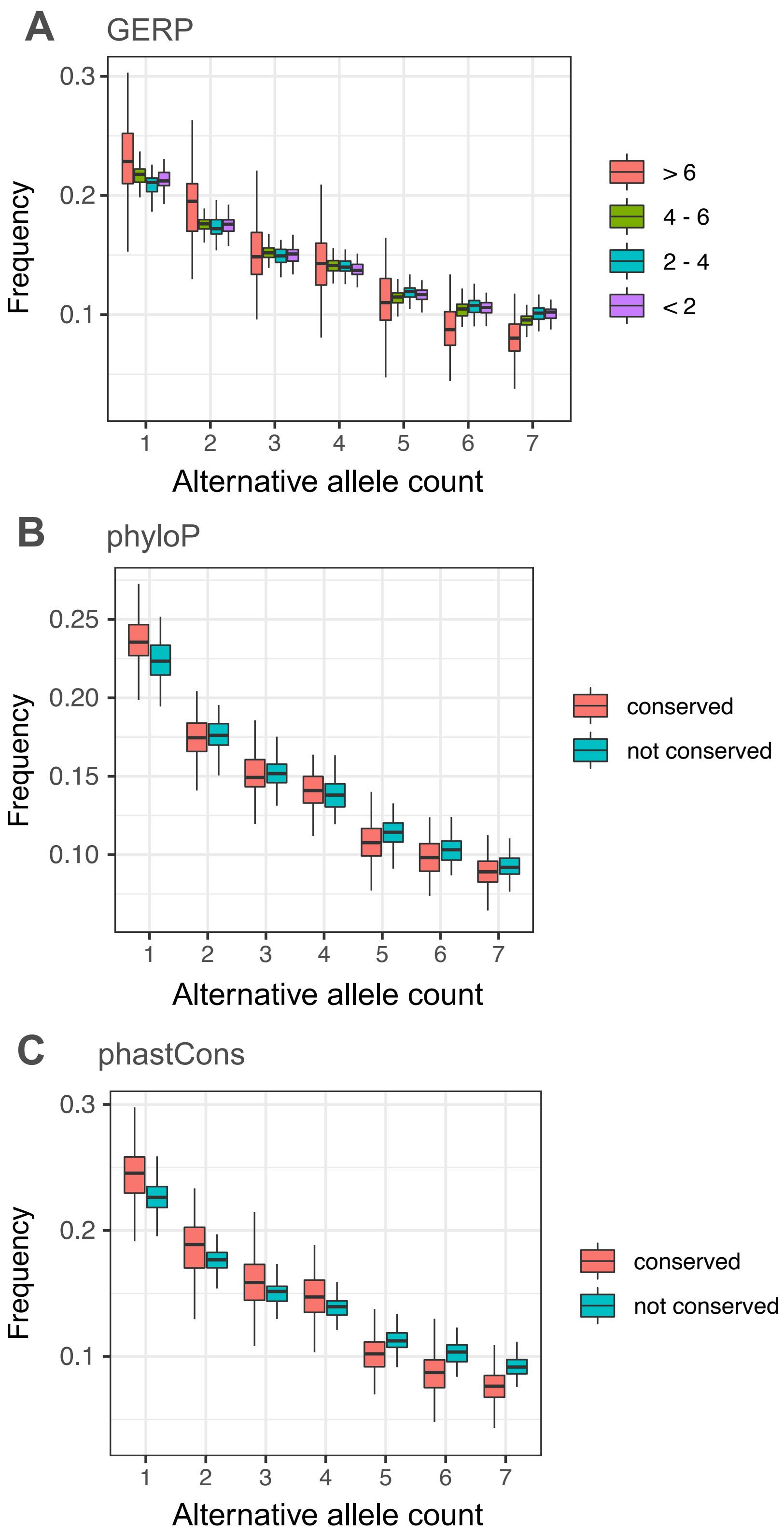

Supplementary Figure 10: Site-frequency spectra (SFS) in Alpine ibex. Categorization of SNPs based on A) GERP scores divided into four ranges, B) phyloP scores (conserved: > 1, not conserved: ≤ 1) and C) phastCons scores (conserved: =1, not conserved: < 1). See Figure 2 for the SFS of SNPs categorized into SnpEff categories high, moderate, low and modifier. Box plot elements as in Supplementary Figure 2A.

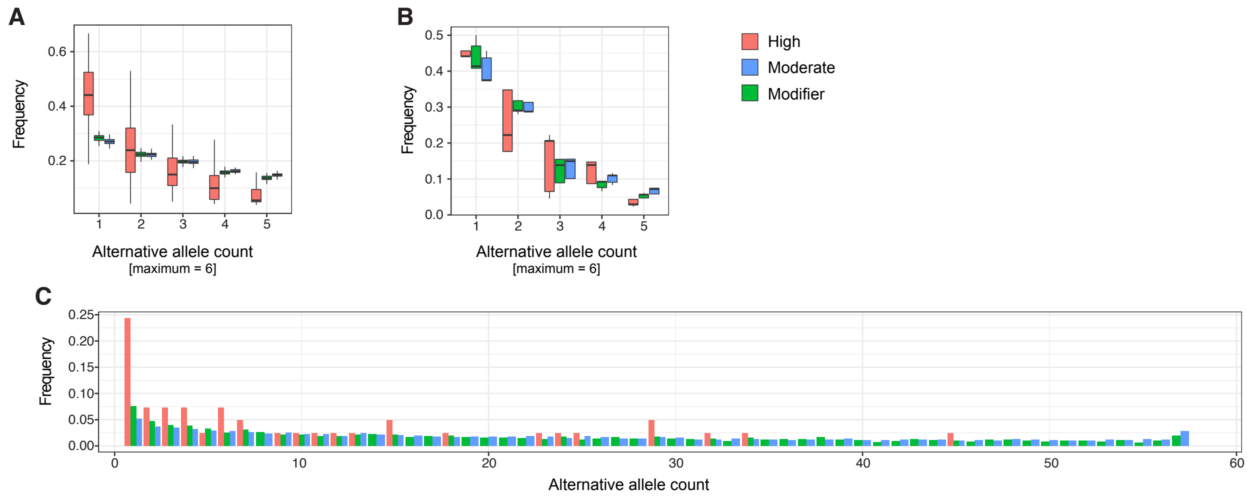

Supplementary Figure 11: Site frequency spectra (SFS) for different categories of SNP mutations. Categories are based on SnpEff (high, moderate and modifier categories). A) SFS downsampled to three Alpine ibex individuals based on jackknifing (100 replicates). B) SFS downsampled to three Iberian ibex individuals based on jackknifing (100 replicates). C) SFS for all Alpine ibex without grouping (see also Figure 2B). Box plot elements as in Supplementary Figure 2A.

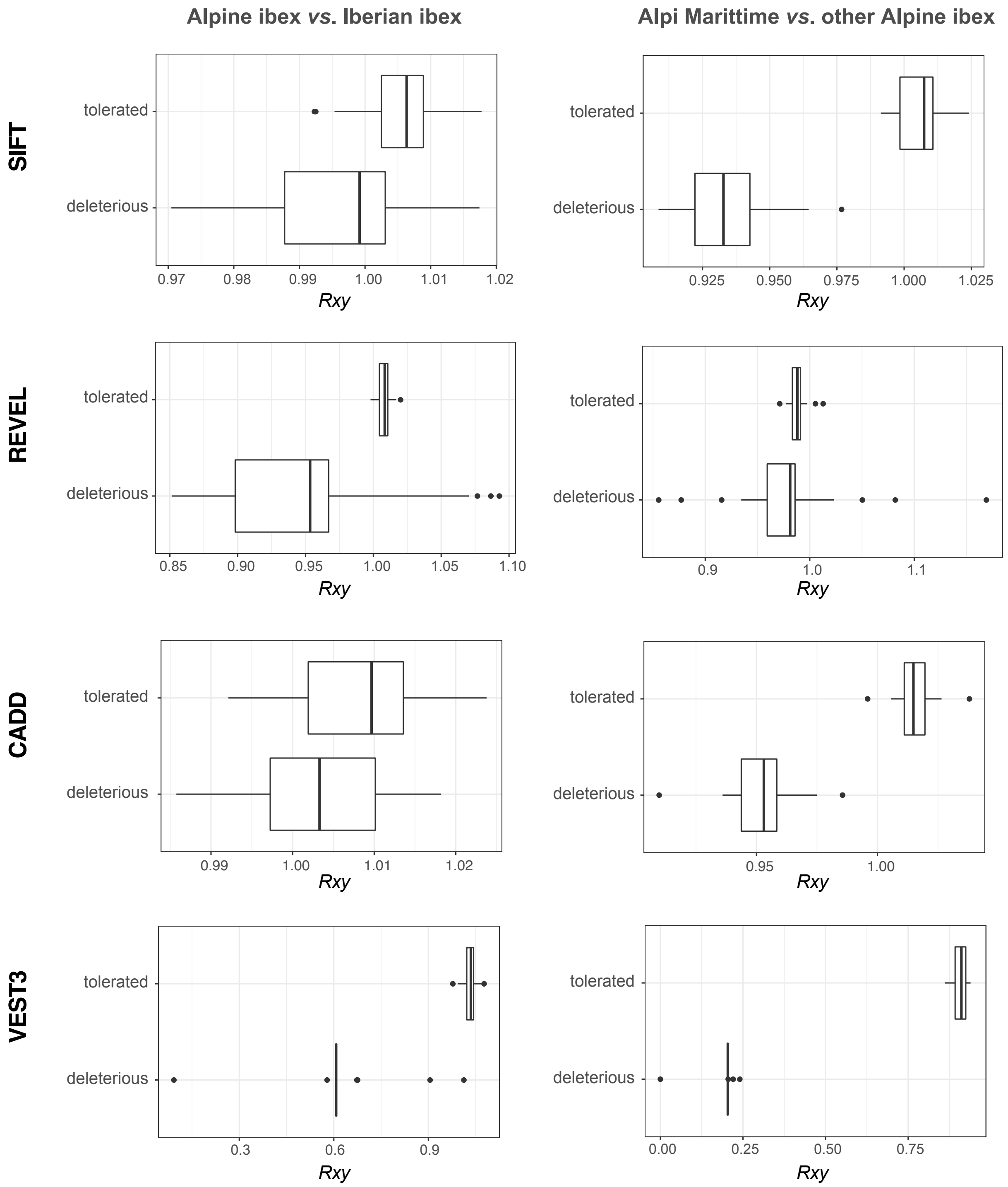

Supplementary Figure 12: *Rxy* analysis. Analyses were performed based on four additional mutation scoring methods (SIFT, Revel, CADD, VEST3). *Rxy* < 1 indicates a relative frequency deficit of the corresponding category in Alpine ibex compared to Iberian ibex (left column) or Alpi Marittime compared to other Alpine ibex (right column). *Rxy* distributions are based on jack-knifing across chromosomes. Score cut-offs used to define deleteriousness were as follows: Revel: 0.75, CADD: 20, SIFT: 0.05, VEST3: 0.91 (99%-percentile). See methods for details. Box plot elements as in Supplementary Figure 2A.

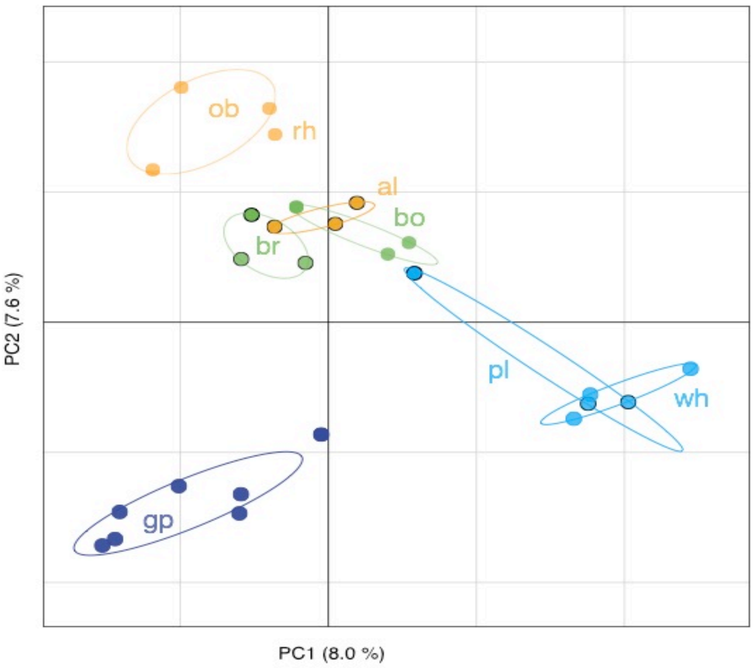

Supplementary Figure 13A: Principal component analysis plot of Alpine ibex. All individuals are included except the individuals from the highly divergent population Alpi Marittime. Circles with black rim indicate the three populations used for all further population reintroductions of Alpine ibex. Same colors of Alpine ibex populations join founder and descendent population. bo, Bire Öschinen; gp, Gran Paradiso; al, Albris; br, Brienzer Rothorn; ob, Oberbauenstock; pl, Pleureur; rh, Rheinwald; wh, Weisshorn.

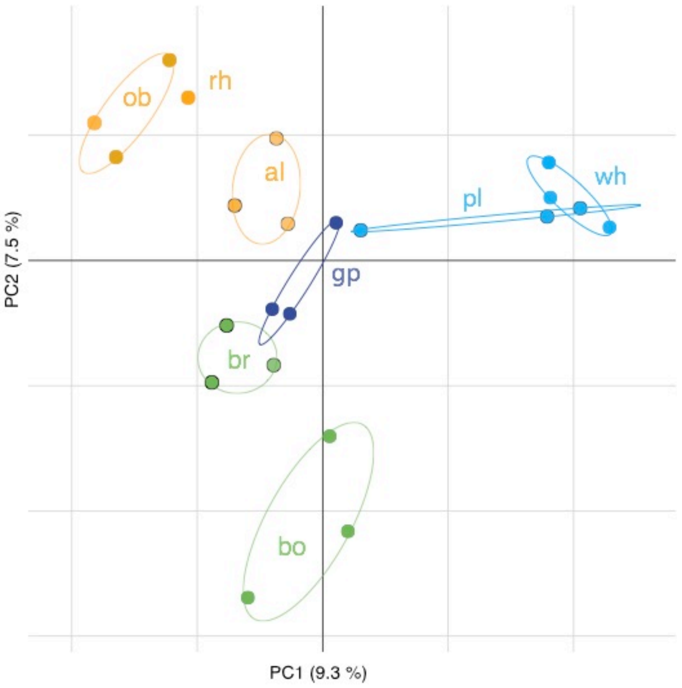

Supplementary Figure 13B: Principal component analysis plot of Alpine ibex. Excluded are the individuals from the highly divergent population Alpi Marittime. The population from Gran Paradiso was reduced to three randomly chosen individuals. bo, Bire Öschinen; gp, Gran Paradiso; al, Albris; br, Brienzer Rothorn; ob, Oberbauenstock; pl, Pleureur; rh, Rheinwald; wh, Weisshorn.

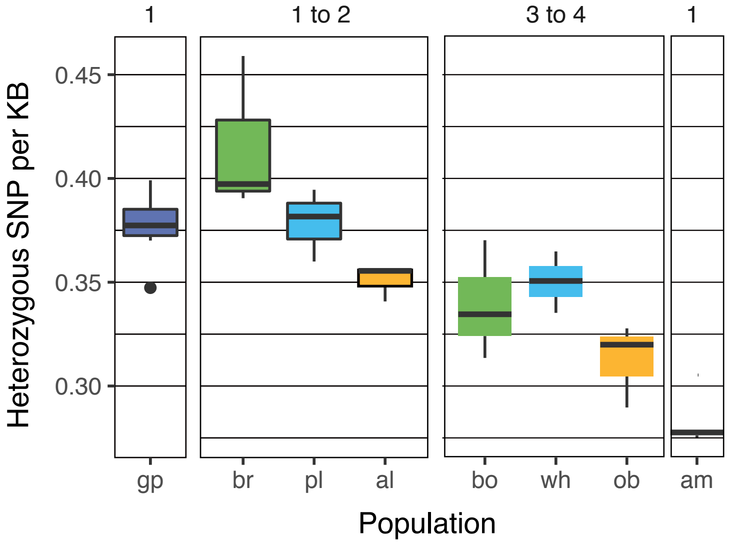

Supplementary Figure 14: Genome-wide heterozygosity per Alpine ibex population. Heterozygosity is expressed as the number of heterozygous, autosomal SNPs per kb. Colors as in Figures 3 and S11. bo, Bire Öschinen; gp, Gran Paradiso; al, Albris; br, Brienzer Rothorn; ob, Oberbauenstock; pl, Pleureur; rh, Rheinwald; wh, Weisshorn. Numbers above panels indicate the number of bottlenecks the corresponding populations underwent during the reintroductions. Box plot elements as in Supplementary Figure 2A.

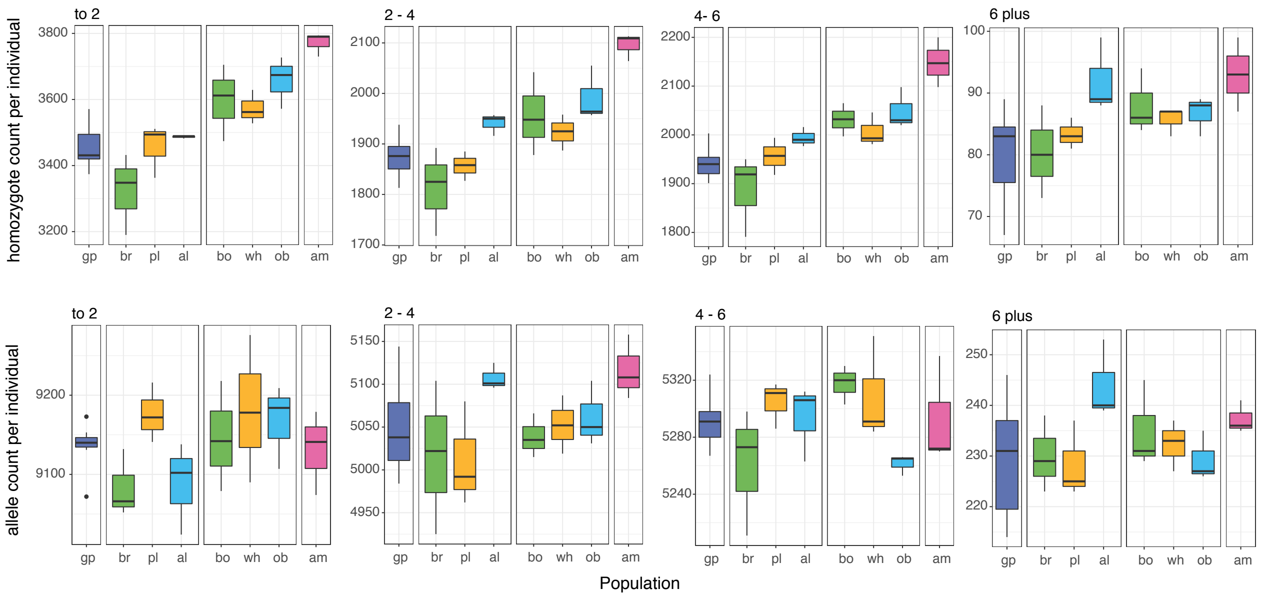

Supplementary Figure 15: Individual mutation load. Individual homozygote counts (upper panel) and allele counts (lower panel) for each Alpine ibex population. Left to right shows GERP score ranges of -2 - 2, 2 - 4, 4 - 6 and >6 of SNP mutations. Colors group Alpine ibex populations into founder and descendent population pairs (see Figure 3). Box plot elements as in Supplementary Figure 2A.

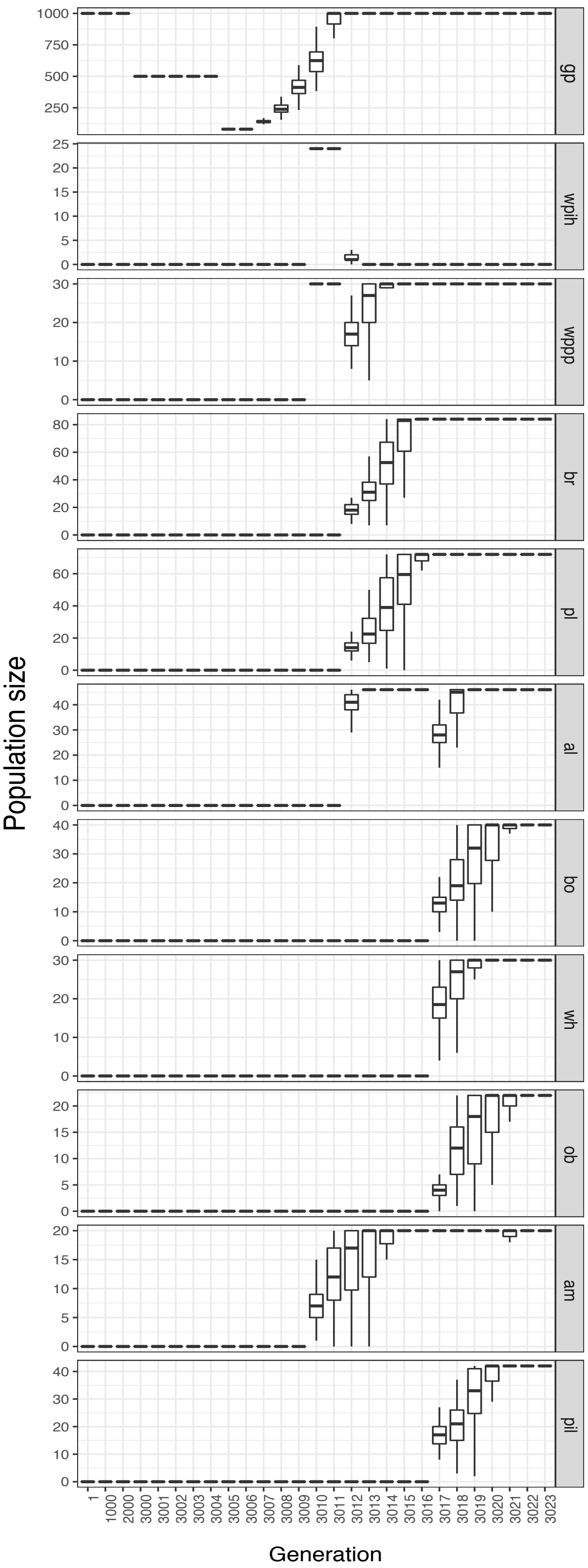

Supplementary Figure 16: Population size changes over the course of the demographic simulations. Population size did not change between generation 1 and 2999. am: Alpi Marittime, gp: Gran Paradiso; ih: Zoo Interlaken Harder; al: Albris; bo: Bire Öschinen; br: Brienzer Rothorn; ob: Oberbauenstock; pl: Pleureur; rh: Rheinwald; wh: Weisshorn; pil: Pilatus; wpih: Wildpark Interlaken Harder; wppp: Wildpark Peter and Paul. Box plot elements as in Supplementary Figure 2A.

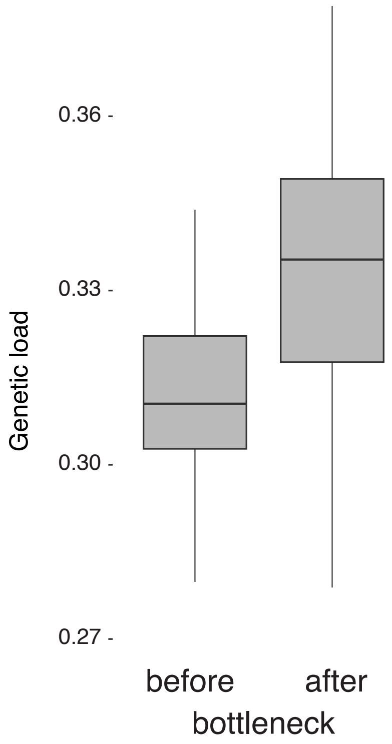

Supplementary Figure 17: Realized genetic load before and after simulated bottleneck. Boxplots showing genetic load before and after the simulated species bottleneck in Alpine ibex. Genetic load was defined as mean individual fitness of females. Box plot elements as in Supplementary Figure 2A.

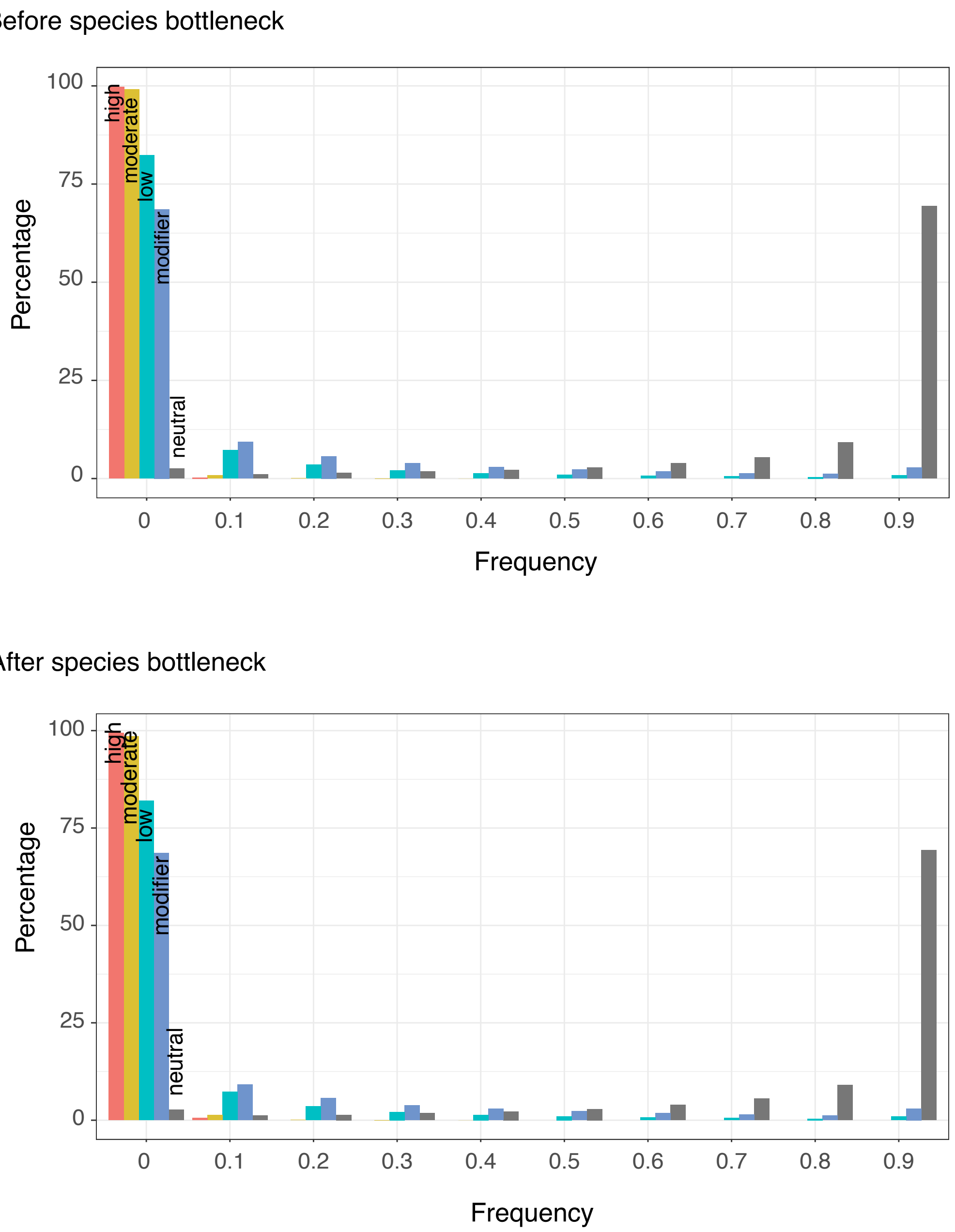

Supplementary Figure 18: Site-frequency spectra before and after simulated bottleneck. Shown are SFS for different mutation categories (neutral, modifier, low, moderate, high). Deleterious mutation categories were defined based on the selection coefficient s as follows: Modifier: $s < 0.0001$, Low: $0.0001 \leq s < 0.01$, Moderate: $0.01 \leq s < 0.1$, High: $0.1 \leq s$. The bottleneck occurred between generations 2999 and 3023.

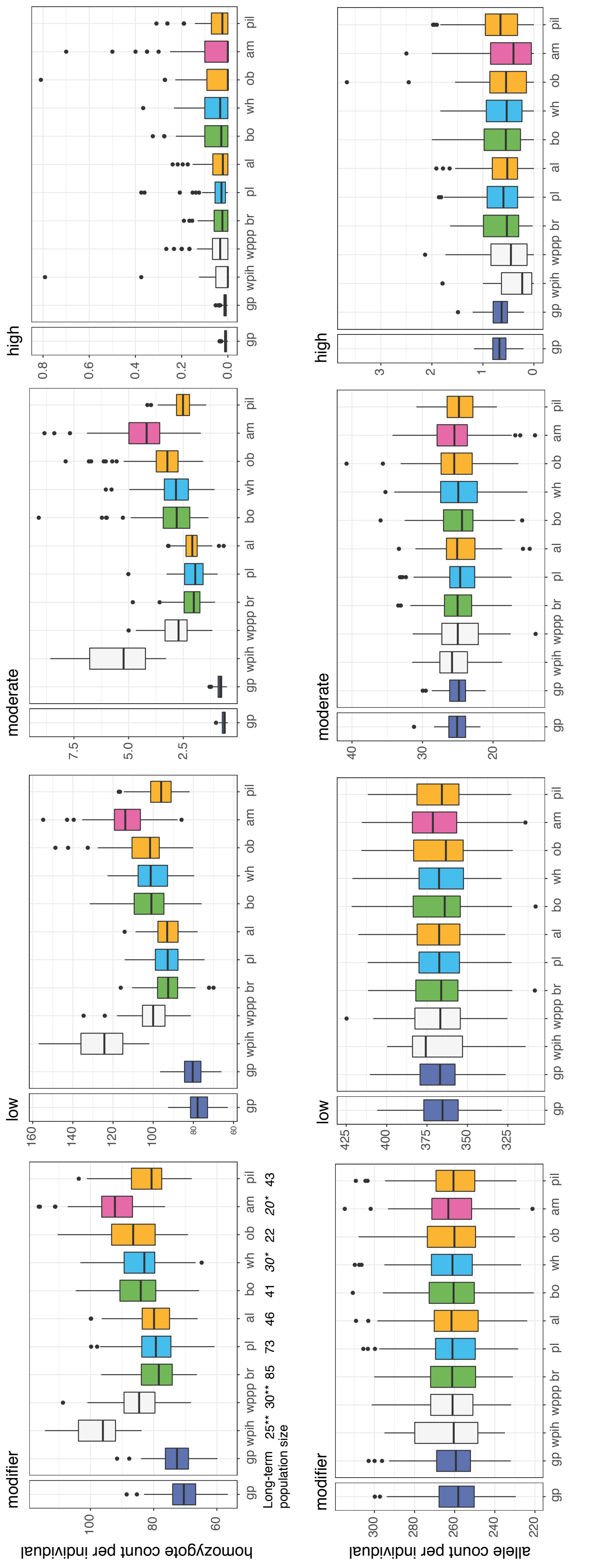

Supplementary Figure 19: Individual genetic load per simulated population. Outcome of the demographic simulations showing individual homozygote and allele counts grouped by population. Simulated populations included all major wild parks and populations that contributed to the founding of the Alpine ibex populations under study (see also Figure 3A). Matching colours group source populations with populations that were founded from them (except for wpih, wppp, gp and am). Deleterious mutation categories were defined based on the selection coefficient *s* as follows: Modifier: $s < 0.0001$, Low: $0.0001 \leq s < 0.01$, Moderate: $0.01 \leq s < 0.1$, High: $0.1 \leq s$. Abbreviations: gp: Gran Paradiso, wpih: Wildpark Interlaken Harder, wppp: Wildpark Peter and Paul, br: Brienzer Rothorn, pl: Pleureur, al: Albris, bo: Bire Oeschinen, wh: Weisshorn, ob: Oberbauenstock, am: Alpi Marittime, pil: Pilatus. Box plot elements as in Supplementary Figure 2A.

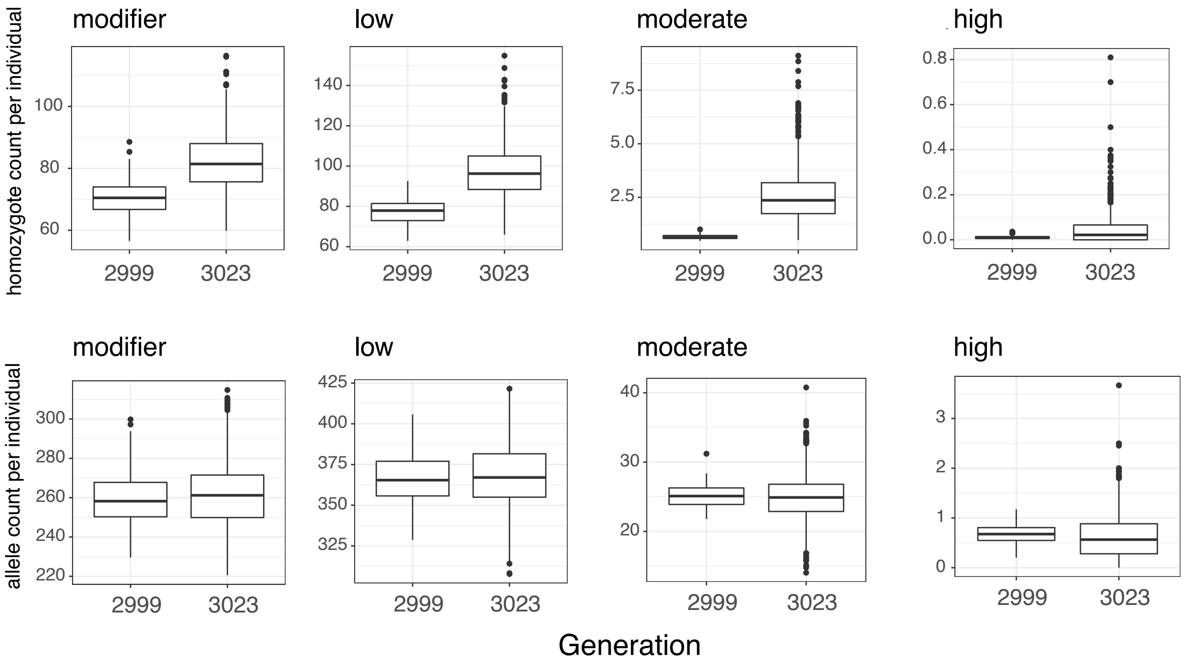

Supplementary Figure 20: Individual genetic mutation load before and after simulated bottlenecks. Outcome of the demographic simulations showing individual homozygote and allele counts before and after the species bottleneck (generations 2999 and 3023, respectively). Deleterious mutation categories were defined based on the selection coefficient s as follows: Modifier: $s < 0.0001$, Low: $0.0001 \leq s < 0.01$, Moderate: $0.01 \leq s < 0.1$, High: $0.1 \leq s$. Box plot elements as in Supplementary Figure 2A.

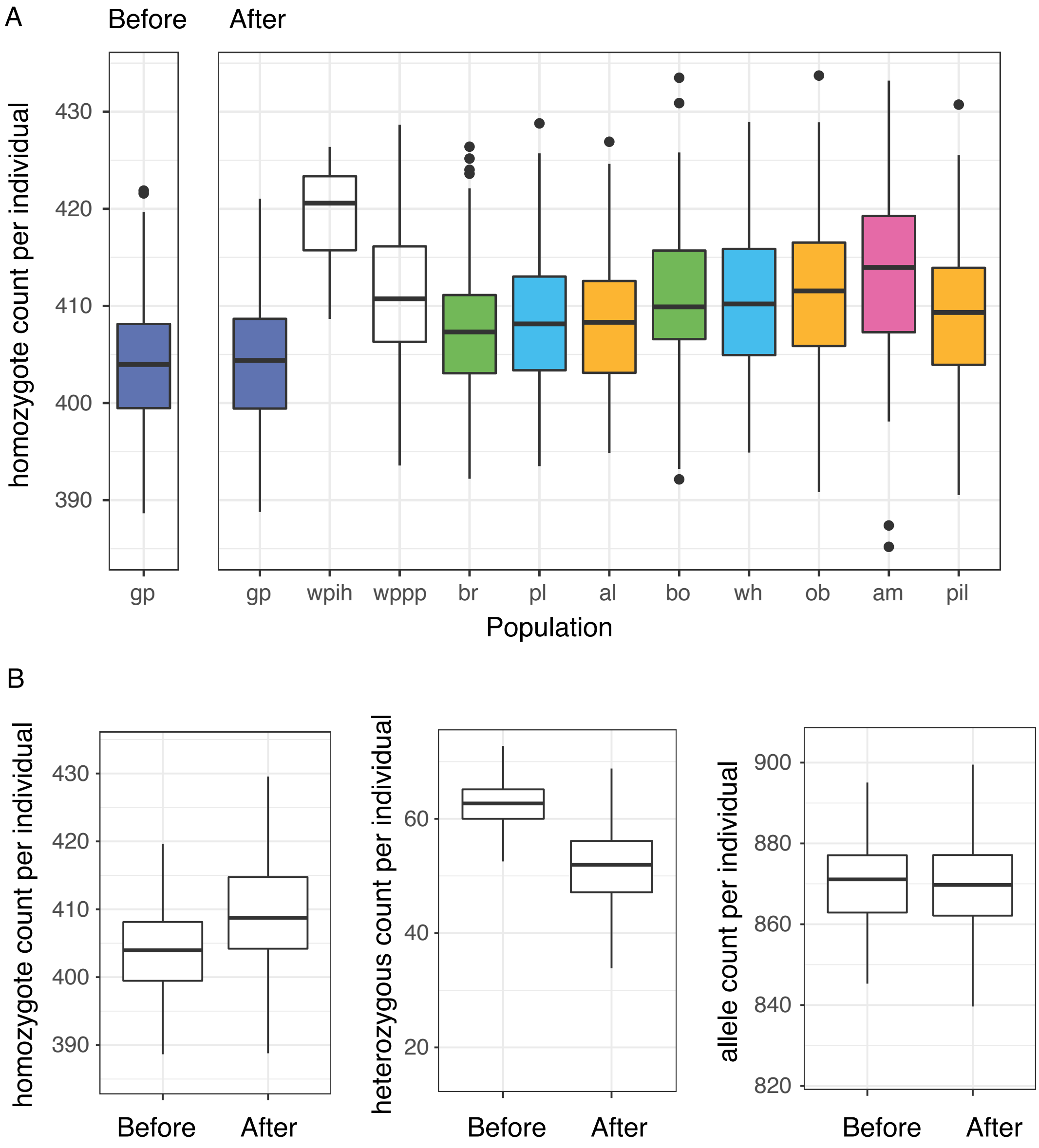

Supplementary Figure 21: Outcome of the demographic simulations at neutral sites. A) Homozygote count for derived neutral alleles before and after the species bottleneck (generations 2999 and 3023, respectively). Simulated populations included all major wild parks and populations that contributed to the founding of the Alpine ibex populations under study (see also Figure 3A). Matching colours group source populations with populations that were founded from them (except for wpih, wppp, gp and am). Abbreviations: gp: Gran Paradiso, wpih: Wildpark Interlaken Harder, wppp: Wildpark Peter and Paul, br: Brienzer Rothorn, pl: Pleureur, al: Albris, bo: Bire Oeschinen, wh: Weisshorn, ob: Oberbauenstock, am: Alpi Marittime, pil: Pilatus. B) Homozygote and heterozygote counts, and allele count per individual of derived neutral alleles before and after the species bottleneck. The category "after" includes all populations for which genomic data was generated with the exception of the wild parks and the Pilatus (pil) population. Box plot elements as in Supplementary Figure 2A.

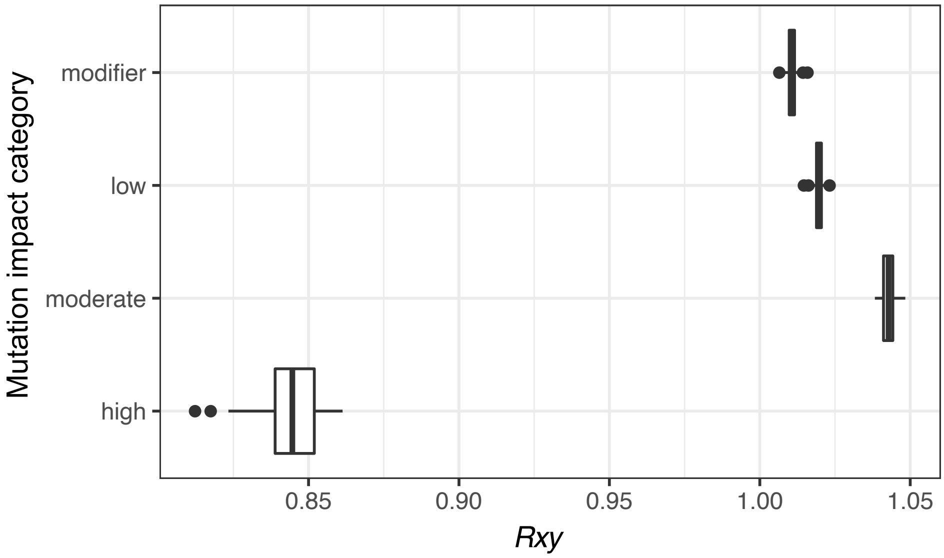

Supplementary Figure 22: ***Rxy* analysis of Alpi Marittime versus Gran Paradiso.** *Rxy* analysis contrasting the strongly bottlenecked Alpi Marittime population with the Gran Paradiso population across the spectrum of SnpEff impact categories. Box plot elements as in Supplementary Figure 2A.

Supplementary Figures 23 – 31: Visualization of SNP variant quality parameters used for filtering. The applied cut-offs are shown with a vertical line.

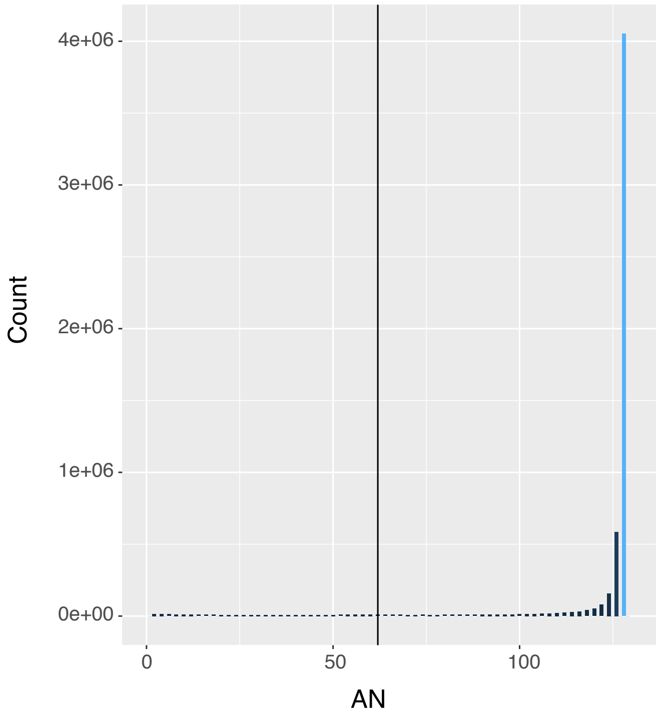

Supplementary Figure 23: Distribution of the AN statistic. Number of called alleles (corresponds to twice the number of samples genotyped at the locus).

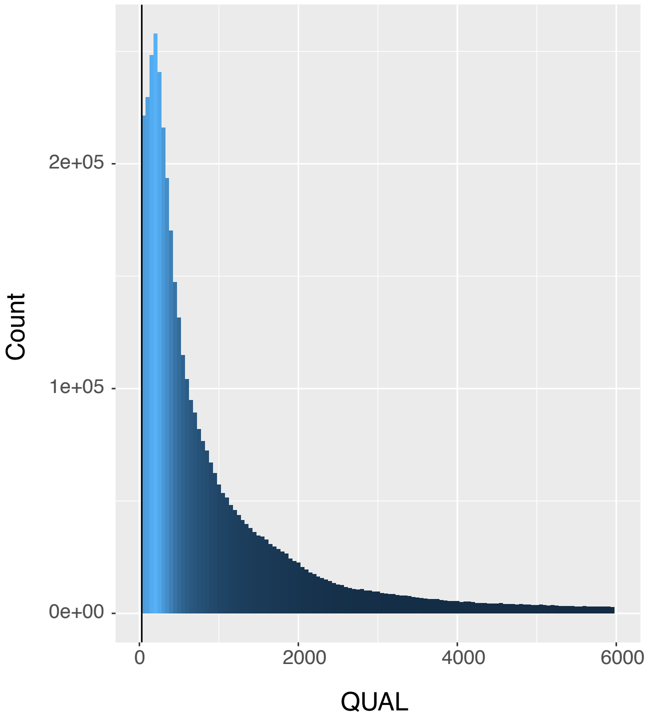

Supplementary Figure 24: Distribution of the overall SNP quality (QUAL) statistic.

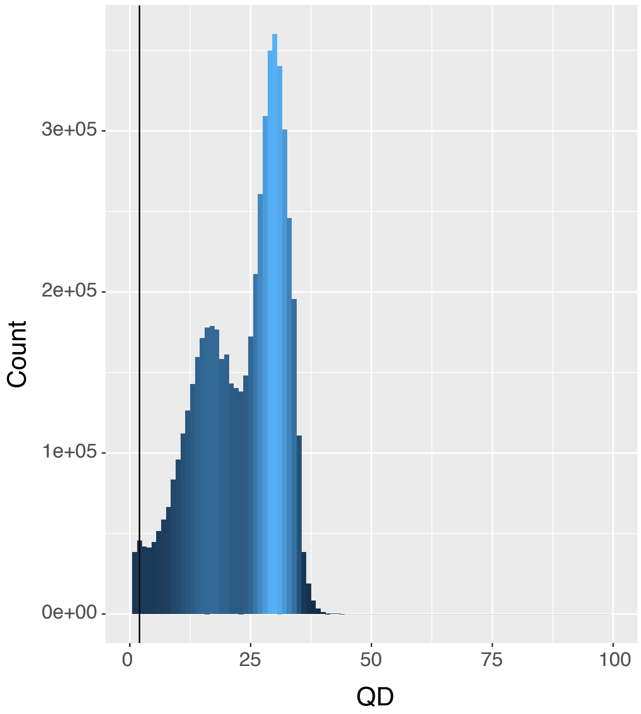

Supplementary Figure 25: Distribution of the QualByDepth (QD) statistic. Confidence of the variant quality (QUAL) divided by the unfiltered depth of the samples called as non-reference.

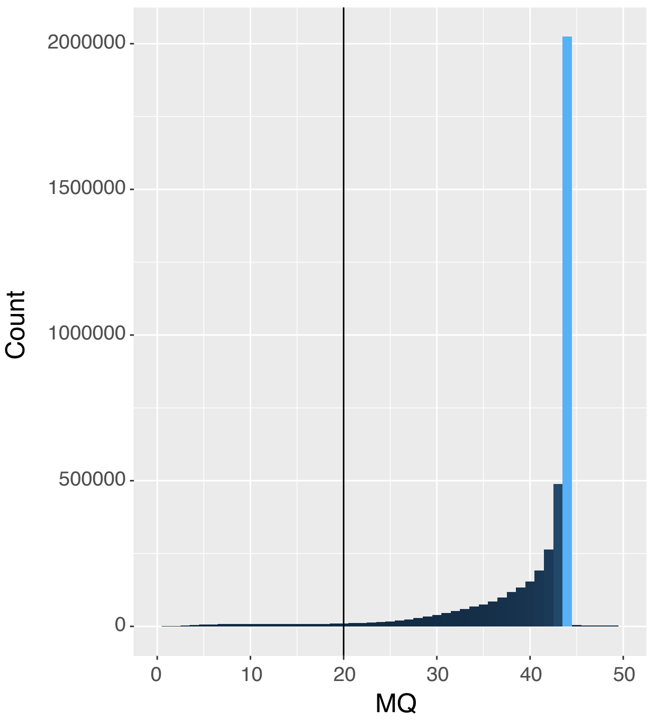

Supplementary Figure 26: Distribution of the RMSMappingQuality (MQ) statistic. Root mean square of the mapping quality calculated across all reads from all samples.

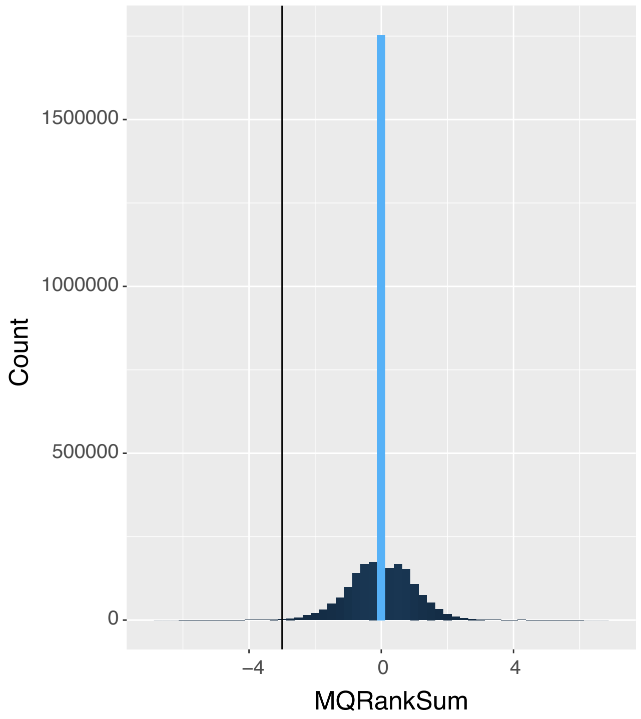

Supplementary Figure 27: Distribution of the MappingQualityRankSumTest (MQRankSum) statistic. A test for differences in mapping quality between reference versus alternate allele.

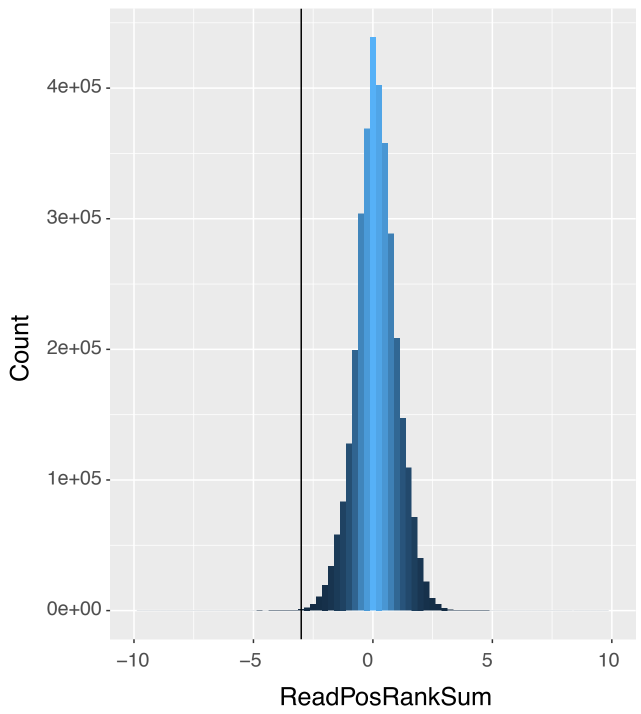

Supplementary Figure 28: Distribution of the ReadPosRankSumTest statistic. A test for the even distribution of variants along reads.

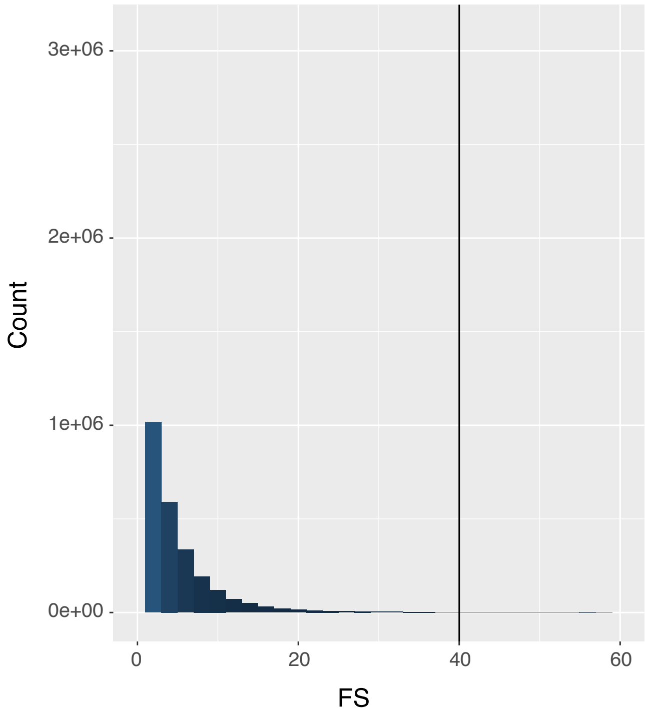

Supplementary Figure 29: Distribution of the FisherStrand (FS) statistic. A test for strand bias, i.e. if a variant is predominantly associated with one of the two read strands (forward or reverse).

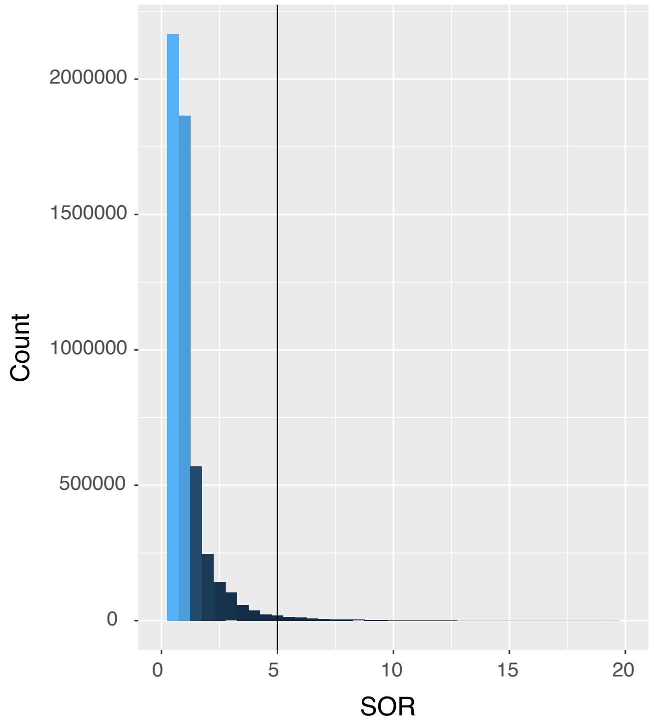

Supplementary Figure 30: Distribution of the StrandOddsRatio (SOR) statistic.

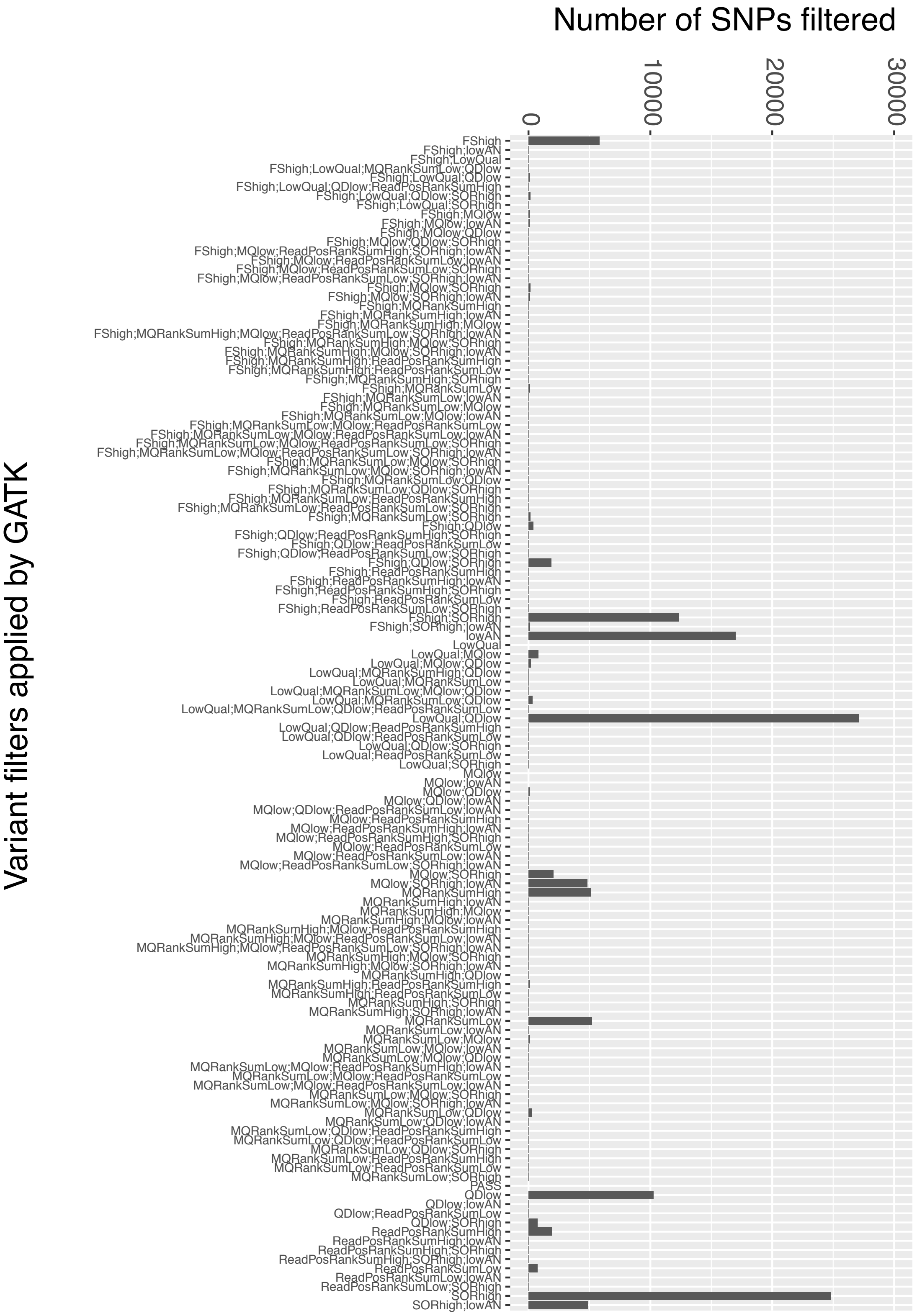

Supplementary Figure 31: Summary of SNP loci filtering outcome. Candidate SNP variants filtered out by different filter combinations. See methods for individual filter settings.

Supplementary Figures 32 - 39: Visualization of indel variant quality parameters used for filtering. The applied cut-offs are shown with a vertical line**.**

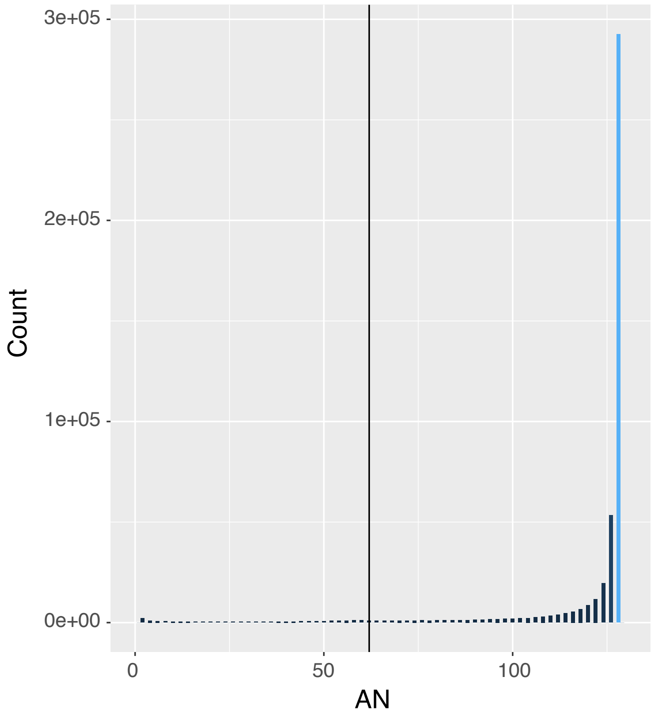

Supplementary Figure 32: Distribution of the AN statistic (indels). Number of called alleles (corresponds to twice the number of samples genotyped at the locus).

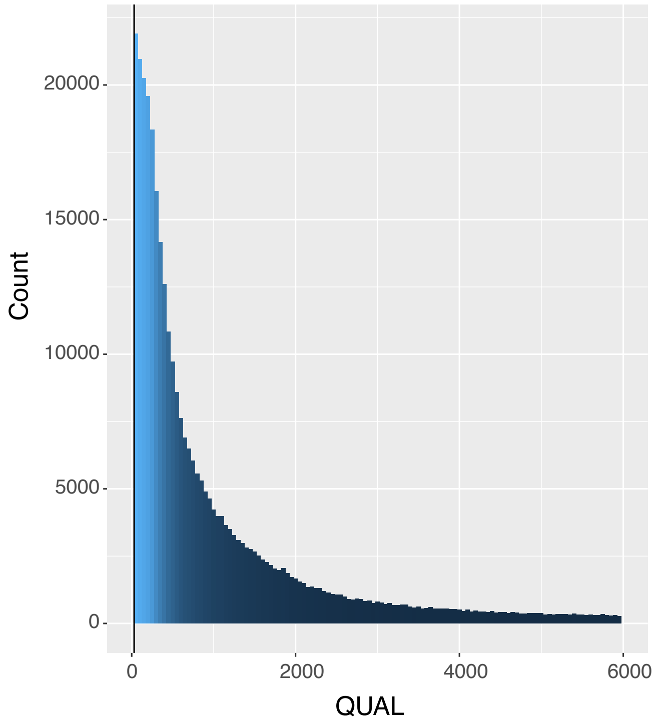

Supplementary Figure 33: Distribution of the overall indel quality (QUAL) statistic.

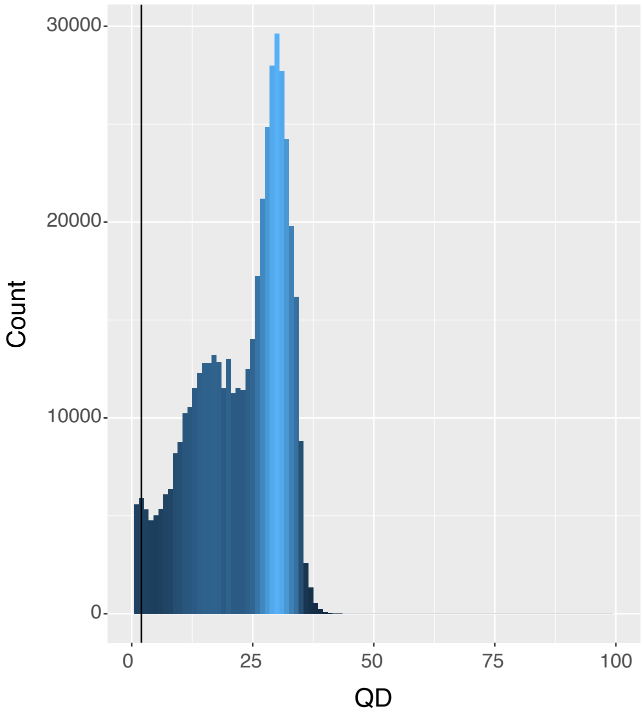

Supplementary Figure 34: Distribution of the QualByDepth (QD) statistic (indels). Confidence of the variant quality (QUAL) divided by the unfiltered depth of the samples called as non-reference.

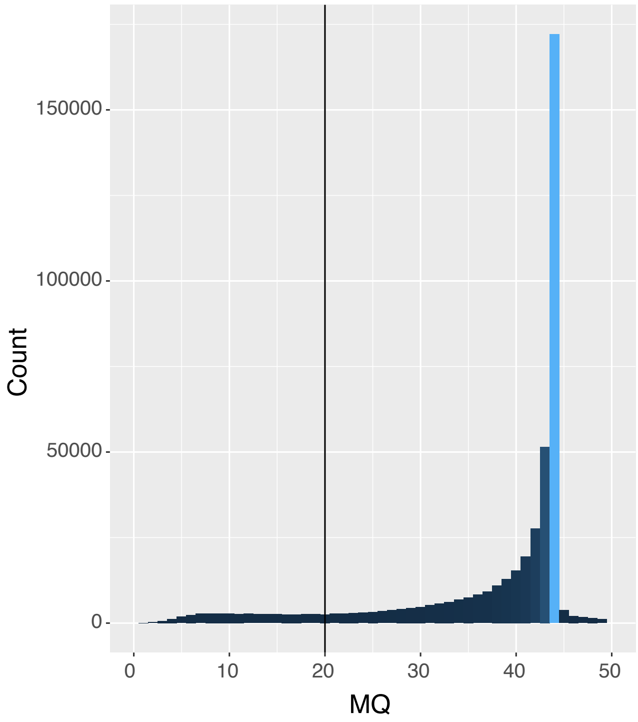

Supplementary Figure 35: Distribution of the RMSMappingQuality (MQ) statistic (indels). Root mean square of the mapping quality calculated across all reads from all samples.

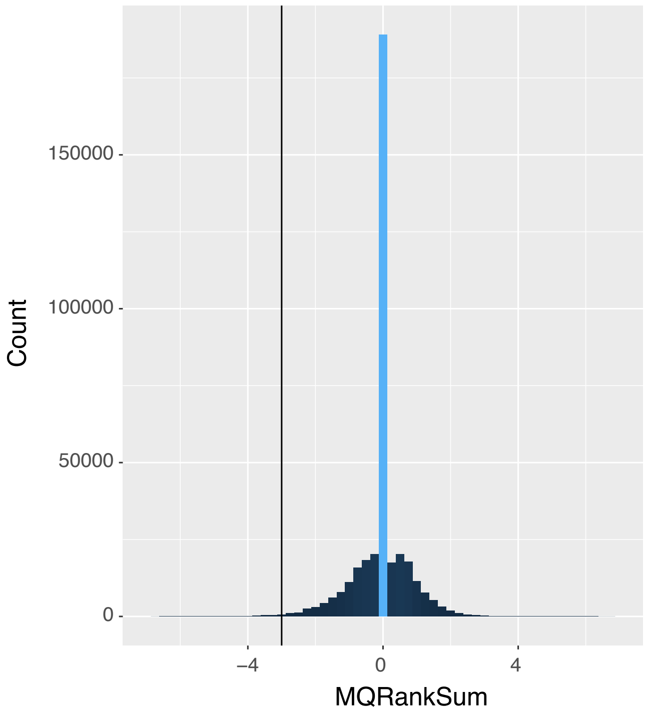

Supplementary Figure 36: Distribution of the MappingQualityRankSumTest (MQRankSum) statistic (indels). A test for differences in mapping quality between reference versus alternate allele.

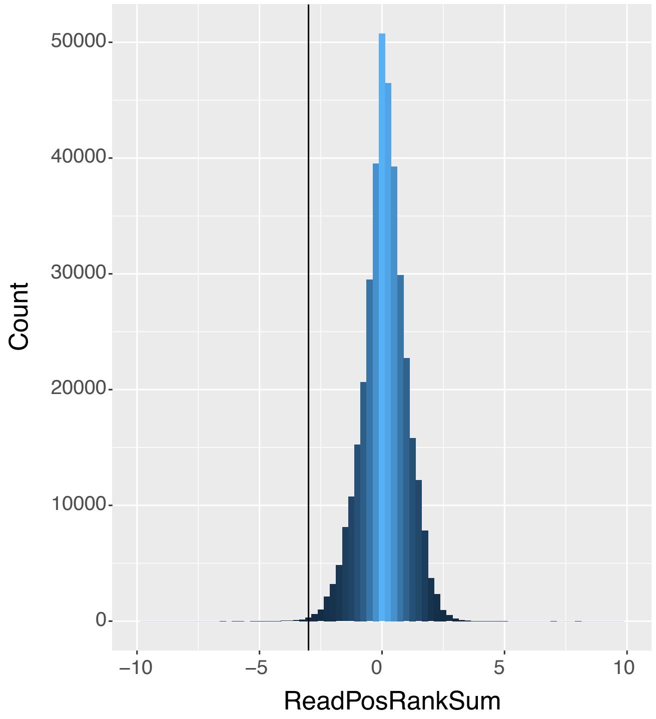

Supplementary Figure 37: Distribution of the ReadPosRankSumTest statistic (indels). A test for the even distribution of variants along reads.

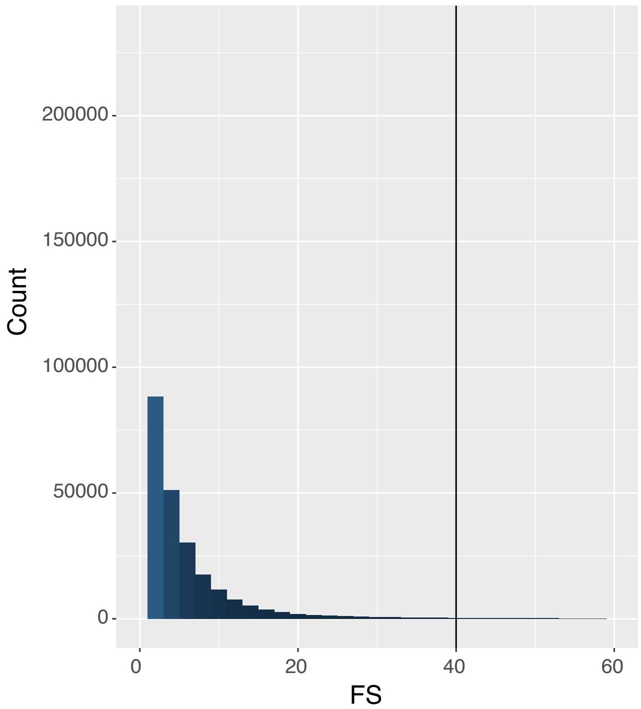

Supplementary Figure 38: Distribution of the FisherStrand (FS) statistic (indels). A test for strand bias, i.e. if a variant is predominantly associated with one of the two read strands (forward or reverse).

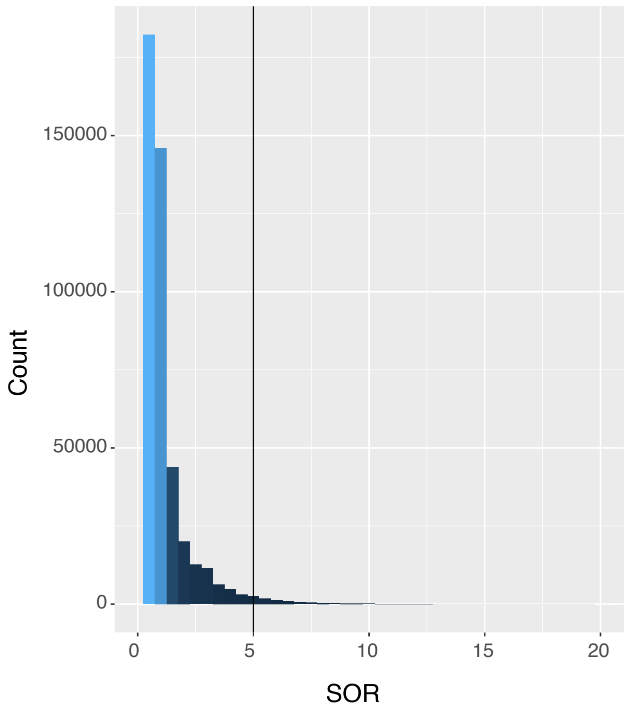

Supplementary Figure 39: Distribution of the StrandOddsRatio (SOR) statistic (indels).

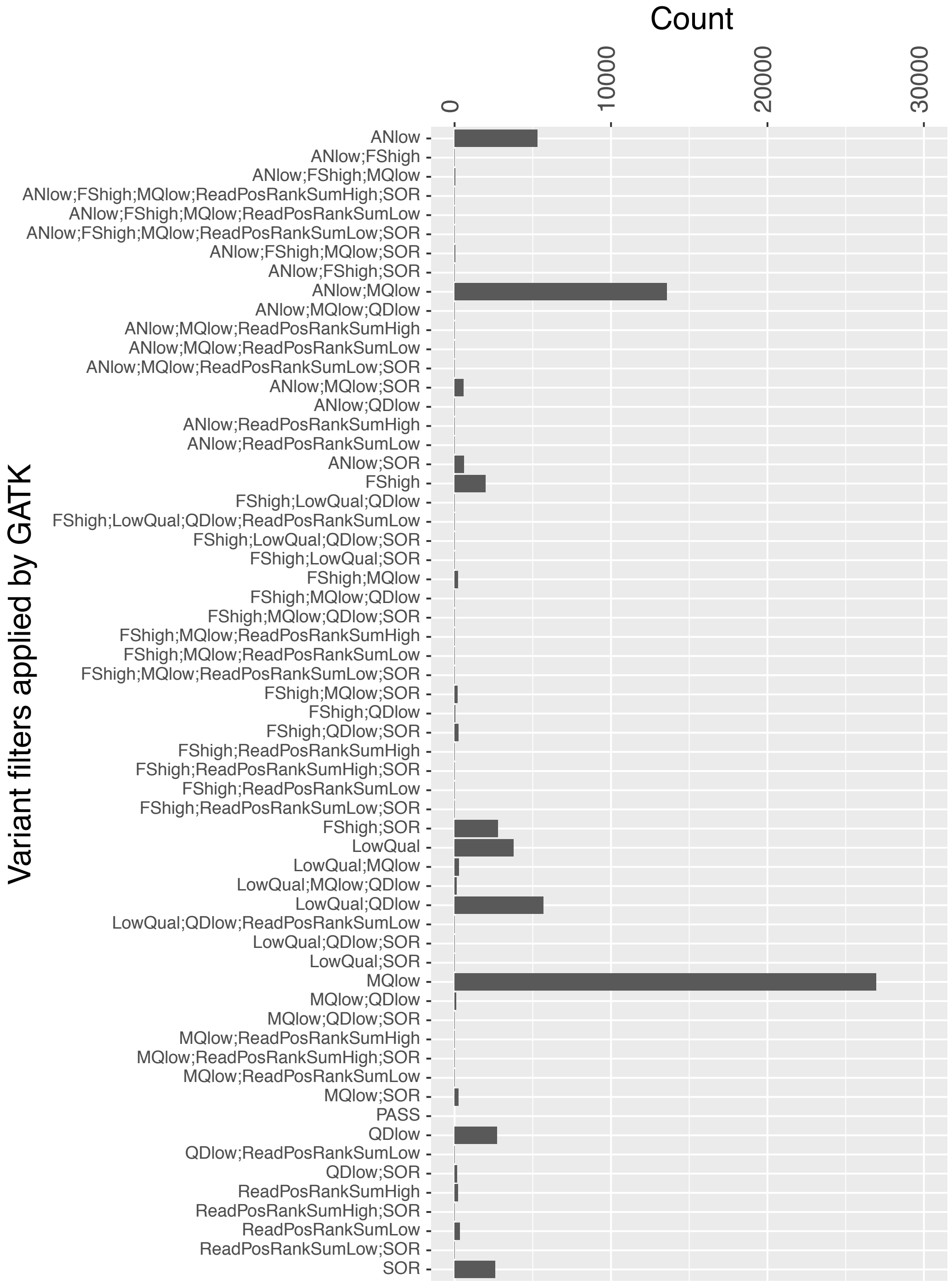

Supplementary Figure 40: Outcome of indel variant filtration. Candidate indel variants filtered out by different filter combinations. See methods for individual filter settings.

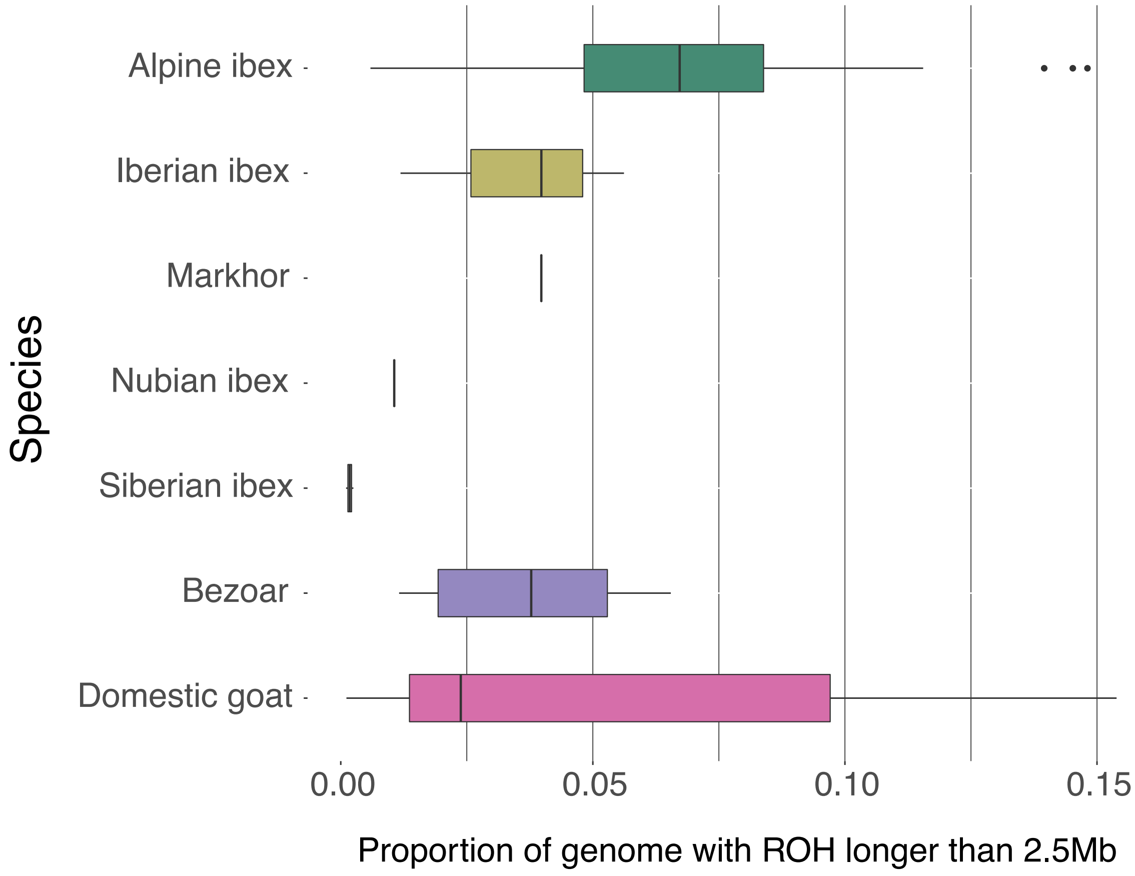

Supplementary Figure 41: Runs of homozygosity (ROH) among species evaluated using PLINK. Proportion of the genome with ROH longer than 2.5 Mb estimated using the software PLINK. Box plot elements as in Supplementary Figure 2A.

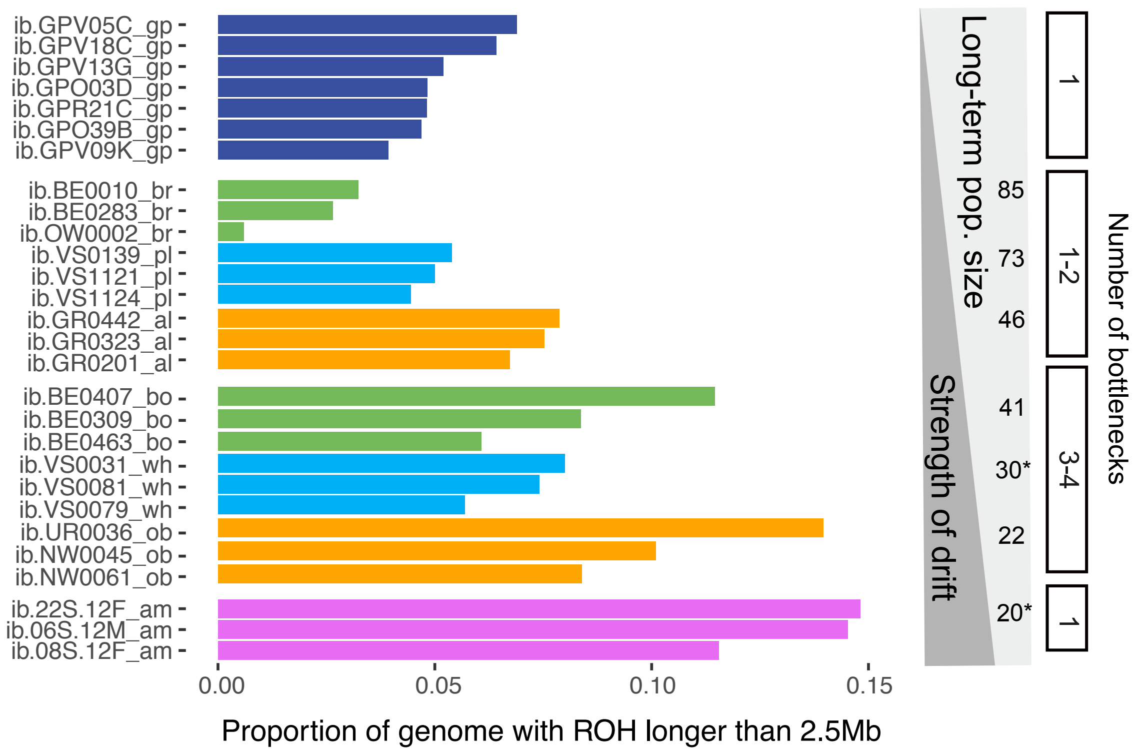

Supplementary Figure 42: Runs of homozygosity (ROH) in Alpine ibex evaluated using PLINK. Proportion of the genome with ROH longer than 2.5 Mb estimated using the software PLINK.

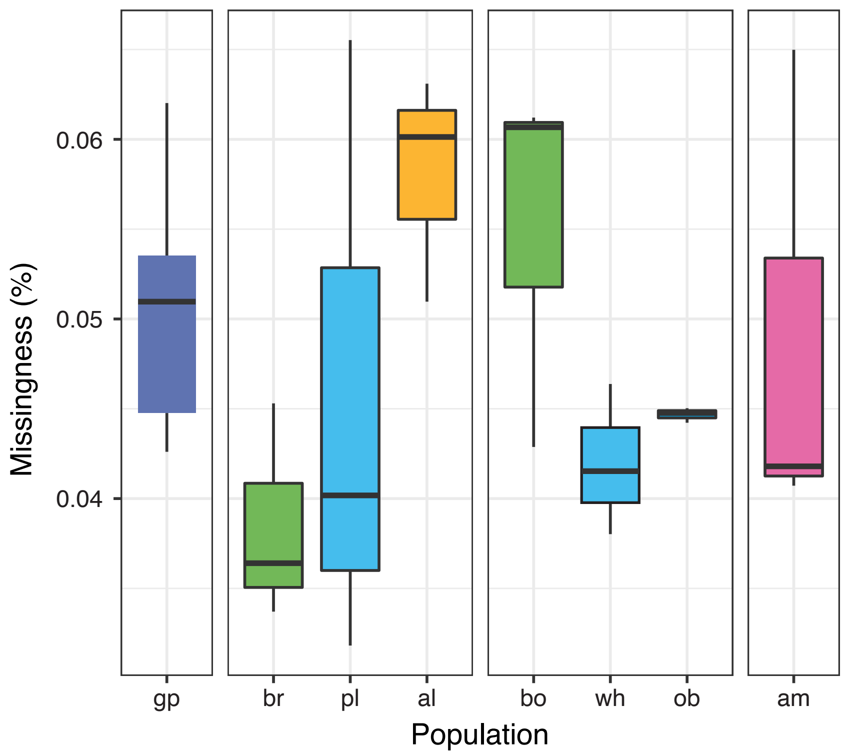

Supplementary Figure 43: Missingness across loci and populations. Boxplot showing the percentage of loci with missing data (missingness) reported by individual and grouped by population. Box plot elements as in Supplementary Figure 2A.

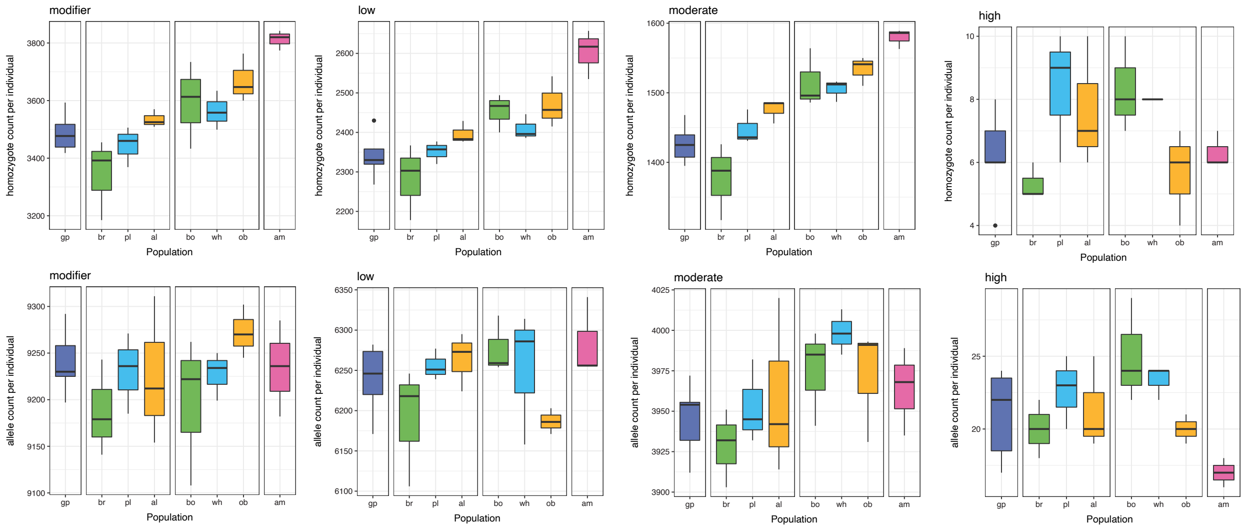

Supplementary Figure 44: Individual genetic load (based on SnpEff). Individual homozygote counts (upper panel) and allele counts (lower panel) for each Alpine ibex population with no missingness allowed. Left to right shows SNP mutation category as predicted by SnpEff (modifier, low, moderate and high). Colors group Alpine ibex populations into founder and descendent population pairs (see also Figures 3 and S13A). Box plot elements as in Supplementary Figure 2A.

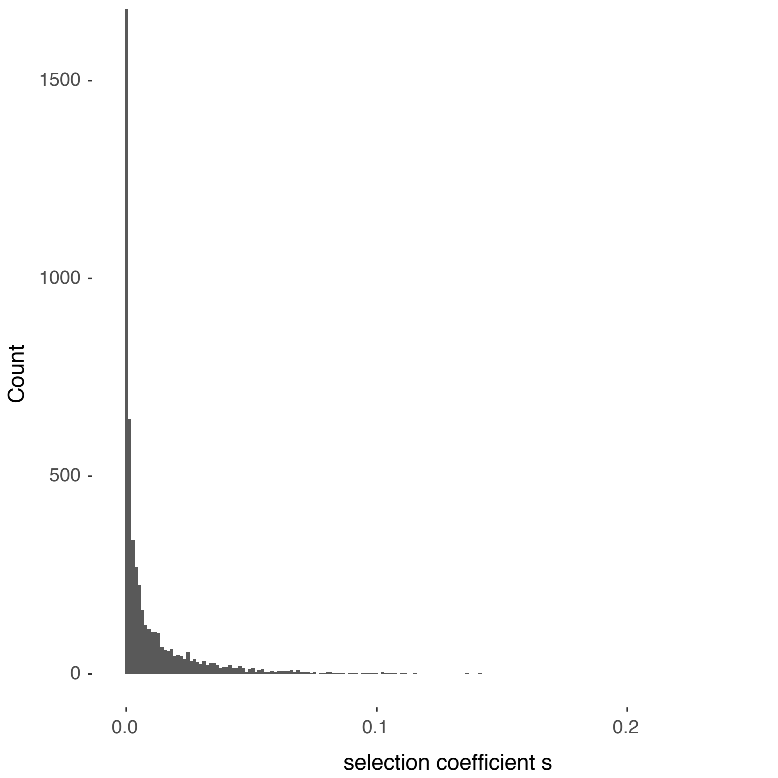

Supplementary Figure 45: Distribution of the selection coefficient *s* used in the simulations.

SUPPLEMENTARY TABLES

Supplementary Table 1A: **Demographic information on Alpine ibex populations.** Gran Paradiso was the only surviving population and at the source of all introduced populations. Effective founder group size differs from total released numbers if knowledge was available about mortality after population establishment. Long-term population size was calculated as the harmonic mean of census size since population founding. *) Estimated numbers based on historic record.

**Alpine ibex** [extant census 50'000 individuals]	**Date of foundation**	**Males released**	**Females released**	**Total released**	**Effective founder group size**	**Long-term population size (from foundation up to 2007)**	**Census 2007**
**Population**							
**Gran Paradiso [1,2]**		-	-	-	100*	n/a	n/a
**Albris (al) [3]**	1920-34	16	26	42	42	46	1033
**Alpi Marittime (am) [4,5]**	1920-30			25	6	20*	750
**Bire Oeschinen (bo) [3]**	1961-62	4	9	13	13	41	68
**Brienzer Rothorn (br) [3]**	1921-25	8	10	18	18	85	288
**Oberbauenstock (ob) [3]**	1968-86	10	12	22	22	22	164
**Pilatus (pi) [3]**	1961-65	8	7	(unknown sex: 2) 17	17	43	104
**Pleureur (pl) [3]**	1928-35	6	9	15	15	73	(year 2003) 806
**Weisshorn (wh) [6]**	1962-69	18	6	24	24	30*	354

Supplementary Table 1B: **Demographic information on three major Iberian ibex populations.** Included are both extant subspecies of *Capra pyrenaica.* Bottleneck periods represent the most dramatic, known reductions in population size. Sierra Nevada and Maestrazgo populations experienced only recent bottlenecks (1960s).

**Iberian ibex**	**Subspecies**	**Strengths of known bottlenecks periods** [estimated individuals]	**Population size changes over time** [if available]	**Current population size**
**Sierra Nevada (sn)**	*Capra pyrenaica hispanica*	~600 (1960s)[7]	n/a	16’000[8]
**Maestrazgo (hb)**	*Capra pyrenaica hispanica*	~30 (1960s)[7]	450 (1966)[8]<br>700 (1970)[8-11]<br>>3000 (1977)[8-11]<br>3800 (1979)[8-11]<br>> 5000 (1981)[8-11]	7000[7]
**Sierra de Gredos (vi)**	*Capra pyrenaica victoriae*	50 (1895)[7]<br>12 (1905)[7]<br>350 (1912)[7]<br>500 (1914)[7]<br>>1000 (1917)[7]	2150 (1961)[7]<br>4000 (1976)[8-10]<br>9000 (1978)[8-10]<br>8000 (1993)[8-10]	8000[7]

Supplementary Table 2: **Overview of individual sampling.** Overview of all sequenced individuals grouped by species including the IUCN conservation status and recent demographic events. Whole-genome sequencing data, reference genome mapping and single nucleotide polymorphism (SNP) statistics are shown.

Sample	Population	Sex	Location	Coordinates (Swiss grid or latitude/longitude)		Raw sequencing data (Gbp)	Mapping rate (%)	Coverage (X)	Number of SNPs genotyped	Number of heterozygote SNPs
**Alpine ibex: Recent bottlenecks, IUCN: Least concern**<br>N=29; 3’539’135 SNPs; nucleotide diversity: 0.00039										
**ib.06S.12M_am**	Alpi Maritimi	male	Rovina			60.0	99.0	17.5	56'126'898	667'046
**ib.08S.12F_am**	Alpi Maritimi	female	Matto			65.2	98.9	18.8	56'126'955	669'730
**ib.22S.12F_am**	Alpi Maritimi	female	Barra			60.2	99.1	17.6	56'126'911	660'179
**ib.BE0010_br**	Brienzer Rothorn	female	Stelli, Hofstetten	181400	649600	64.3	99.2	19.5	56'127'089	938'208
**ib.BE0283_br**	Brienzer Rothorn	female	Salenwang	180600	650250	69.5	99.1	21.1	56'127'180	954'605
**ib.BE0309_bo**	Bire Oeschinen	female	Schwarzhorn Oeschinen	151400	624850	59.6	98.7	18.1	56'126'958	803'869
**ib.BE0407_bo**	Bire Oeschinen	male	Oeschinensee, Heuberg	150750	621800	55.5	99.1	17.2	56'126'864	753'333
**ib.BE0463_bo**	Bire Oeschinen	male	Oeschinensee, Underbärgli	150750	622600	59.7	99.1	18.7	56'126'974	889'571
**ib.GPO03D_gp**	Gran Paradiso	male	Orco, Chiapili sup	45.4684	7.1474	58.8	98.9	17.3	56'126'869	906'666
**ib.GPO39B_gp**	Gran Paradiso	male	Orco	45.4644	7.1588	58.3	99.0	17.2	56'126'868	958'991
**ib.GPR21C_gp**	Gran Paradiso	male	Rhemes	45.5637	7.1400	57.2	99.2	17.7	56'126'849	925'983
**ib.GPV05C_gp**	Gran Paradiso	female	Valsaveranche, Levionaz	45.5821	7.2262	60.9	99.2	18.4	56'126'978	834'684
**ib.GPV09K_gp**	Gran Paradiso	male	Valsaveranche, Levionaz	45.5746	7.2398	56.4	99.0	17.0	56'126'827	924'811
**ib.GPV13G_gp**	Gran Paradiso	male	Valsaveranche, Levionaz	45.5779	7.2333	56.8	99.0	16.6	56'126'804	900'629
**ib.GPV18C_gp**	Gran Paradiso	male	Valsaveranche, Levionaz	45.5779	7.2333	63.5	99.1	20.0	56'127'046	889'186
**ib.GR0140_rh**	Rheinwaldhorn	female	Scalutta Gem Safien	166250	741820	54.3	98.7	16.8	56'126'827	733'524
**ib.GR0201_al**	Albris	female	Munt Blais, Gm S-chanf	166065	799210	56.1	99.2	17.4	56'126'937	854'091
**ib.GR0323_al**	Albris	female	Paradies Suot, Pontresina	150740	791325	60.8	99.1	18.3	56'126'987	856'238
**ib.GR0442_al**	Albris	male	Val da Fain Tschüffer, Pontresina	150160	797230	60.2	99.0	18.6	56'127'038	818'724
**ib.NW0045_ob**	Oberbauerstock	male	Hoh Brisen	194450	678200	55.6	98.9	17.3	56'126'878	768'646
**ib.NW0061_ob**	Oberbauerstock	female	Niederbauen	200200	685050	58.9	99.2	17.8	56'126'939	787'534
**ib.OW0002_br**	Brienzer Rothorn	male	Eisee, Giswil	182251	646972	63.8	98.6	19.6	56'127'113	1'102'820
**ib.UR0036_ob**	Oberbauerstock	female	IsenthalBärenstock	193371	681175	61.0	97.8	18.1	56'126'939	695'986
**ib.VS0031_wh**	Weisshorn	female	Jungen; St. Nklaus	115700	627300	56.5	99.1	17.4	56'126'843	842'412
**ib.VS0079_wh**	Weisshorn	female	Seetal, Gde. Grächen	117000	627750	62.3	99.1	20.2	56'127'095	876'610
**ib.VS0081_wh**	Weisshorn	female	Jungen, Gde. St. Niklaus	117000	627750	60.0	99.0	19.5	56'126'935	805'493
**ib.VS0139_pl**	Pleureur	male	Dixènce-Evolène	101000	595000	62.0	99.0	19.4	56'127'046	916'987
**ib.VS1121_pl**	Pleureur	female	Verbier	105100	586700	60.9	98.9	18.4	56'126'971	864'945
**ib.VS1124_pl**	Pleureur	male	Boussine	87250	592900	54.4	98.8	17.0	56'126'830	947'985
**Iberian ibex: Recent bottlenecks, IUCN: Least concern**<br>N=4; 4’353’580 SNPs; nucleotide diversity: 0.00066										
**py.V53_vi**			Sierra de Gredos			60.0	91.3	15.3	56'126'235	1'274'362
**py.V66_vi**			Sierra de Gredos			60.3	94.2	15.3	56'126'292	1'000'193
**py.M518_sn**	Sierra Nevada		Sierra Nevada			88.0	99.1	26.9	56'127'272	1'000'228
**py.Z5_hb**			Maestrazgo			56.4	99.2	17.0	56'126'788	960'783
**Nubian ibex: Fragmented populations, IUCN: Vulnerable**<br>N=2, 6’980’112 SNPs, nucleotide diversity: 0.00151										
**nu.ibx20**			Sinai, Egypt			102.5	98.6	17.1	56'126'494	3'131'909
**nu.ibx61**			Howtat, Central Saudi Arabia			46.9	98.7	18.4	56'126'885	2'536'433
**Markhor: Fragmented populations, IUCN: Near Threatened**<br>N=1, 1’988’577 SNPs, nucleotide diversity: 0.00077										
**fal.Cafal1**			Dasht-i Jum Reserve, Tajikistan			148.7	98.9	38.6	56'127'403	1'845'346
**Siberian ibex: Large populations, IUCN: Least concern**<br>N=2, 10’433’689 SNPs, nucleotide diversity: 0.00224										
**si.Casi1**		male	South of Issyk lake			57.4	98.7	15.8	56'126'287	4'379'849
**si.Casi2**		male	Dasht-i Jum Reserve, Tajikistan			80.1	98.8	22.0	56'126'976	4'823'262
**Bezoar: Large populations, IUCN: Vulnerable**<br>N=6, 15’182’016 SNPs, nucleotide diversity: 0.00191										
**IRCA-C3-1001**		male	Marakan	45.16	38.63	37.6	99.1	11.5	56'127'088	3'212'565
**IRCA-G2-5063**		male	Arasbaran	47.13	39.18	36.5	98.7	11.6	56'127'115	3'610'951
**IRCA-I11-0001**		male	Khan Gormaz	48.20	34.60	19.1	99.0	5.9	56'126'263	2'293'061
**IRCA-I6-5237**		male	Karnagh	48.39	37.16	34.6	99.1	11.3	56'127'111	3'402'754
**IRCA-K12-0005**		male	Lashgar Dar	49.05	34.21	19.7	99.0	6.3	56'126'443	2'524'631
**IRCA-M12-0008**		male	khomein	50.34	34.18	32.4	99.0	10.1	56'127'049	3'972'538
**Domestic goat**<br>N=16, 21’943’431 SNPs, nucleotide diversity: 0.00198										
**FRCH-AL-0002**	Alpine	female	.	-0.36	46.69	41.5	99.2	12.8	56'127'200	3'826'421
**FRCH-SA-0001**	Saanen	female	.	-0.28	46.57	40.4	99.2	12.1	56'127'136	3'919'140
**IRCH-B3-5031**	.	female	Khoiy	44.94	38.65	38.0	99.2	12.0	56'127'181	4'539'659
**IRCH-C5-5206**	.	male	Nushin Shahr	45.06	37.78	39.0	99.1	12.1	56'127'214	3'170'212
**IRCH-F3-5044**	.	female	Ahar	46.85	38.52	39.4	99.2	11.6	56'127'110	4'051'773
**IRCH-F4-5093**	.	female	Vazarghan	46.61	38.30	39.6	99.2	11.9	56'127'128	3'322'150
**IRCH-G5-5185**	.	male	Sarab	47.08	37.97	35.5	99.1	11.5	56'127'172	4'358'311
**ITCH-SA-0001**	Saanen	.	.	.	.	46.7	98.9	13.8	56'127'216	3'999'955
**ITCH-SA-0005**	Saanen	.	.	.	.	44.5	98.4	13.0	56'127'156	3'983'967
**MOCH-AA10-2195**	landrace	male	Maaterka	-1.82	32.52	35.8	99.0	11.2	56'127'031	3'895'474
**MOCH-L10-3100**	landrace	male	Ayir	-9.06	32.70	38.3	99.1	12.0	56'127'148	2'717'970
**MOCH-Q15-1143**	landrace	female	Foum Zguid	-6.58	30.08	37.3	99.2	11.7	56'127'163	2'416'000
**MOCH-R5-0037**	landrace	female	Khmiss Sahel	-6.09	35.20	35.1	99.1	10.9	56'127'117	3'544'065
**MOCH-S8-2252**	landrace	male	Al Hajeb	-5.67	33.81	41.1	99.1	13.0	56'127'171	2'596'402
**MOCH-V5-3059**	landrace	female	Beni Boufrah	-4.30	35.10	40.8	99.2	12.6	56'127'190	4'151'251
**hi.PCGA06**	Peacock	.	.	.	.	62.3	99.2	19.5	56'127'329	4'098'566

Supplementary Table 3: Summary of runs of homozygosity (ROH). Total length per ROH category. Note that categories are overlapping. Populations abbreviated by am: Alpi Marittime, gp: Gran Paradiso; al: Albris; bo: Bire Öschinen; br: Brienzer Rothorn; ob: Oberbauenstock; pl: Pleureur; rh: Rheinwald; wh: Weisshorn. NA: no population/breed assigned. A) Analysis settings af.dflt 0.4, M=0.0000012, run with Viterbi. B) Settings af.dflt 0.4, M=0.0000012.

Individual	100-500 Kb	>100 kb	>500 kb	>2.5 Mb	2.5-10 Mb	>10 Mb	Population/ Breed	Species
	Settings: af.dflt 0.4, M=0.0000012, with Viterbi							
**ib.GR0201_al**	617'316'226	1'499'036'113	881'719'887	278'054'595	278'054'595	0	al	*C. ibex*
**ib.GR0323_al**	648'739'982	1'568'056'539	919'316'557	271'420'074	271'420'074	0	al	*C. ibex*
**ib.GR0442_al**	638'635'462	1'619'373'279	980'737'817	293'579'234	293'579'234	0	al	*C. ibex*
**ib.08S.12F_am**	550'622'660	1'883'994'916	1'333'372'256	580'980'545	569'620'719	11'359'826	am	*C. ibex*
**ib.06S.12M_am**	526'257'448	1'814'566'314	1'288'308'866	531'521'579	531'521'579	0	am	*C. ibex*
**ib.22S.12F_am**	485'235'630	1'826'304'781	1'341'069'151	604'013'148	604'013'148	0	am	*C. ibex*
**ib.BE0309_bo**	624'378'681	1'625'733'750	1'001'355'069	325'649'131	314'522'057	11'127'074	bo	*C. ibex*
**ib.BE0407_bo**	529'902'205	1'628'035'706	1'098'133'501	444'950'345	444'950'345	0	bo	*C. ibex*
**ib.BE0463_bo**	714'827'140	1'586'641'537	871'814'397	233'878'805	233'878'805	0	bo	*C. ibex*
**ib.BE0010_br**	743'914'314	1'653'878'572	909'964'258	238'950'631	238'950'631	0	br	*C. ibex*
**ib.BE0283_br**	808'881'190	1'775'794'639	966'913'449	264'644'590	254'642'196	10'002'394	br	*C. ibex*
**ib.OW0002_br**	761'260'031	1'667'152'587	905'892'556	199'762'116	199'762'116	0	br	*C. ibex*
**ib.GPR21C_gp**	678'473'743	1'434'407'783	755'934'040	175'346'650	175'346'650	0	gp	*C. ibex*
**ib.GPV05C_gp**	650'121'652	1'594'127'217	944'005'565	266'705'862	266'705'862	0	gp	*C. ibex*
**ib.GPV18C_gp**	777'624'987	1'682'216'743	904'591'756	236'158'730	236'158'730	0	gp	*C. ibex*
**ib.GPO03D_gp**	664'865'678	1'520'317'013	855'451'335	227'583'908	227'583'908	0	gp	*C. ibex*
**ib.GPO39B_gp**	709'970'926	1'471'650'951	761'680'025	151'298'231	151'298'231	0	gp	*C. ibex*
**ib.GPV09K_gp**	662'015'971	1'492'528'666	830'512'695	167'263'444	167'263'444	0	gp	*C. ibex*
**ib.GPV13G_gp**	647'435'577	1'493'675'120	846'239'543	194'612'790	194'612'790	0	gp	*C. ibex*
**ib.NW0045_ob**	573'664'491	1'615'991'379	1'042'326'888	370'839'466	360'195'918	10'643'548	ob	*C. ibex*
**ib.NW0061_ob**	595'069'740	1'616'525'100	1'021'455'360	336'713'979	325'950'950	10'763'029	ob	*C. ibex*
**ib.UR0036_ob**	561'427'932	1'762'789'124	1'201'361'192	514'766'948	504'014'248	10'752'700	ob	*C. ibex*
**ib.VS0139_pl**	746'261'010	1'630'720'974	884'459'964	225'660'318	225'660'318	0	pl	*C. ibex*
**ib.VS1121_pl**	634'868'136	1'645'766'132	1'010'897'996	326'843'120	326'843'120	0	pl	*C. ibex*
**ib.VS1124_pl**	636'991'188	1'368'015'802	731'024'614	139'861'308	139'861'308	0	pl	*C. ibex*
**ib.GR0140_rh**	543'598'033	1'597'032'136	1'053'434'103	324'940'186	324'940'186	0	rh	*C. ibex*
**ib.VS0079_wh**	738'754'492	1'697'873'813	959'119'321	273'245'245	249'102'537	24'142'708	wh	*C. ibex*
**ib.VS0081_wh**	656'387'409	1'725'855'351	1'069'467'942	333'444'887	333'444'887	0	wh	*C. ibex*
**ib.VS0031_wh**	635'312'736	1'548'163'493	912'850'757	301'607'030	291'518'068	10'088'962	wh	*C. ibex*
**nu.ibx20**	355'804'025	398'512'218	42'708'193	0	0	0	NA	*C. nubiana*
**nu.ibx61**	386'976'121	803'470'296	416'494'175	130'605'348	130'605'348	0	NA	*C. nubiana*
**py.Z5_hb**	391'166'242	1'262'581'533	871'415'291	281'367'004	256'892'451	24'474'553	hb	*C. pyrenaica*
**py.M518_sn**	950'904'052	2'037'382'478	1'086'478'426	303'317'928	303'317'928	0	sn	*C. pyrenaica*
**py.V53_vi**	579'842'010	1'102'731'313	522'889'303	47'817'848	47'817'848	0	vi	*C. pyrenaica*
**py.V66_vi**	508'832'531	1'164'493'864	655'661'333	117'445'961	117'445'961	0	vi	*C. pyrenaica*
**si.Casi1**	129'673'268	265'145'849	135'472'581	42'751'180	42'751'180	0	NA	*C. sibirica*
**si.Casi2**	387'043'290	544'294'224	157'250'934	15'395'187	15'395'187	0	NA	*C. sibirica*
**IRCA-C3-1001**	116'433'824	606'632'709	490'198'885	268'128'032	256'267'102	11'860'930	NA	*C. aegagrus*
**IRCA-G2-5063**	146'599'124	364'865'275	218'266'151	65'684'795	65'684'795	0	NA	*C. aegagrus*
**IRCA-I11-0001**	82'755'412	828'874'800	746'119'388	374'898'069	363'284'902	11'613'167	NA	*C. aegagrus*
**IRCA-I6-5237**	103'888'535	592'035'177	488'146'642	199'925'786	199'925'786	0	NA	*C. aegagrus*
**IRCA-K12-0005**	89'520'127	765'249'396	675'729'269	325'751'977	325'751'977	0	NA	*C. aegagrus*
**IRCA-M12-0008**	99'873'735	310'410'173	210'536'438	36'558'518	36'558'518	0	NA	*C. aegagrus*
**fal.Cafal1**	573'621'042	2'314'024'081	1'740'403'039	549'883'291	448'500'903	101'382'388	NA	*C. falconeri*
**FRCH-AL-0002**	111'890'971	322'924'190	211'033'219	92'718'720	92'718'720	0	Alpine	*C. hircus*
**IRCH-B3-5031**	53'738'058	60'736'586	6'998'528	0	0	0	NA	*C. hircus*
**IRCH-C5-5206**	71'091'645	786'587'520	715'495'875	450'008'904	427'535'728	22'473'176	NA	*C. hircus*
**IRCH-F3-5044**	59'526'367	299'946'902	240'420'535	120'980'706	120'980'706	0	NA	*C. hircus*
**IRCH-F4-5093**	57'578'725	696'091'487	638'512'762	371'049'415	358'859'987	12'189'428	NA	*C. hircus*
**IRCH-G5-5185**	72'654'707	87'703'744	15'049'037	0	0	0	NA	*C. hircus*
**MOCH-AA10-2195**	90'182'118	271'781'073	181'598'955	107'678'451	92'861'325	14'817'126	landrace	*C. hircus*
**MOCH-L10-3100**	106'103'980	981'754'026	875'650'046	447'547'432	426'912'917	20'634'515	landrace	*C. hircus*
**MOCH-Q15-1143**	94'753'992	1'103'076'736	1'008'322'744	639'531'078	587'996'693	51'534'385	landrace	*C. hircus*
**MOCH-R5-0037**	76'703'976	484'745'657	408'041'681	245'431'572	233'806'996	11'624'576	landrace	*C. hircus*
**MOCH-S8-2252**	96'022'567	1'028'257'715	932'235'148	552'459'778	510'277'731	42'182'047	landrace	*C. hircus*
**MOCH-V5-3059**	104'574'015	171'184'148	66'610'133	13'521'240	13'521'240	0	landrace	*C. hircus*
**hi.PCGA06**	281'436'611	410'435'725	128'999'114	73'150'162	73'150'162	0	Peacock	*C. hircus*
**FRCH-SA-0001**	100'896'581	244'944'909	144'048'328	71'406'178	61'185'213	10'220'965	Saanen	*C. hircus*
**ITCH-SA-0001**	112'477'470	244'286'695	131'809'225	54'227'488	54'227'488	0	Saanen	*C. hircus*
**ITCH-SA-0005**	104'291'210	215'165'572	110'874'362	39'345'718	39'345'718	0	Saanen	*C. hircus*

Individual	100-500 Kb	>100 kb	>500 kb	>2.5 Mb	2.5-10 Mb	>10 Mb	Population/ Breed	Species
	Settings: af.dflt 0.4, M=0.0000012, without Viterbi							
**ib.GR0201_al**	783'649'374	1'995'664'565	1'212'015'191	450'312'926	429'182'212	21'130'714	al	*C. ibex*
**ib.GR0323_al**	771'984'838	2'074'769'627	1'302'784'789	493'636'590	482'683'987	10'952'603	al	*C. ibex*
**ib.GR0442_al**	744'636'342	2'110'321'145	1'365'684'803	531'212'709	507'408'809	23'803'900	al	*C. ibex*
**ib.08S.12F_am**	506'440'668	2'215'138'383	1'708'697'715	886'398'299	757'676'854	128'721'445	am	*C. ibex*
**ib.06S.12M_am**	535'367'583	2'182'318'972	1'646'951'389	843'786'402	769'462'039	74'324'363	am	*C. ibex*
**ib.22S.12F_am**	504'894'041	2'177'064'831	1'672'170'790	924'503'363	788'231'278	136'272'085	am	*C. ibex*
**ib.BE0309_bo**	731'956'217	2'104'565'569	1'372'609'352	567'146'789	519'862'556	47'284'233	bo	*C. ibex*
**ib.BE0407_bo**	682'427'109	2'062'002'272	1'379'575'163	673'696'514	593'848'718	79'847'796	bo	*C. ibex*
**ib.BE0463_bo**	766'167'748	2'112'275'126	1'346'107'378	443'543'188	421'161'453	22'381'735	bo	*C. ibex*
**ib.BE0010_br**	746'218'372	2'155'341'482	1'409'123'110	431'098'700	420'768'536	10'330'164	br	*C. ibex*
**ib.BE0283_br**	648'107'833	2'231'657'101	1'583'549'268	543'880'783	505'040'297	38'840'486	br	*C. ibex*
**ib.OW0002_br**	725'021'312	2'177'987'516	1'452'966'204	477'831'355	454'859'576	22'971'779	br	*C. ibex*
**ib.GPR21C_gp**	876'341'653	2'008'306'378	1'131'964'725	363'481'599	352'877'217	10'604'382	gp	*C. ibex*
**ib.GPV05C_gp**	772'343'629	2'102'615'900	1'330'272'271	453'794'181	441'906'799	11'887'382	gp	*C. ibex*
**ib.GPV18C_gp**	729'634'379	2'175'335'815	1'445'701'436	434'715'929	424'683'247	10'032'682	gp	*C. ibex*
**ib.GPO03D_gp**	784'511'145	2'060'500'449	1'275'989'304	379'316'440	340'167'171	39'149'269	gp	*C. ibex*
**ib.GPO39B_gp**	828'638'496	2'037'365'688	1'208'727'192	316'277'004	316'277'004	0	gp	*C. ibex*
**ib.GPV09K_gp**	814'803'919	2'028'835'721	1'214'031'802	354'293'490	354'293'490	0	gp	*C. ibex*
**ib.GPV13G_gp**	791'183'333	2'006'827'487	1'215'644'154	342'628'909	342'628'909	0	gp	*C. ibex*
**ib.NW0045_ob**	683'383'101	2'055'828'846	1'372'445'745	595'495'883	564'218'995	31'276'888	ob	*C. ibex*
**ib.NW0061_ob**	722'075'711	2'094'887'534	1'372'811'823	622'284'739	573'736'535	48'548'204	ob	*C. ibex*
**ib.UR0036_ob**	617'068'750	2'158'114'200	1'541'045'450	767'497'546	697'638'277	69'859'269	ob	*C. ibex*
**ib.VS0139_pl**	758'740'285	2'145'084'721	1'386'344'436	449'421'486	415'295'277	34'126'209	pl	*C. ibex*
**ib.VS1121_pl**	731'045'337	2'127'252'036	1'396'206'699	534'826'955	469'751'317	65'075'638	pl	*C. ibex*
**ib.VS1124_pl**	873'505'705	1'924'745'798	1'051'240'093	275'850'892	275'850'892	0	pl	*C. ibex*
**ib.GR0140_rh**	669'983'274	2'001'957'025	1'331'973'751	575'091'026	550'389'677	24'701'349	rh	*C. ibex*
**ib.VS0079_wh**	734'275'706	2'152'755'412	1'418'479'706	502'302'612	454'745'847	47'556'765	wh	*C. ibex*
**ib.VS0081_wh**	685'673'558	2'142'824'732	1'457'151'174	579'985'076	541'409'137	38'575'939	wh	*C. ibex*
**ib.VS0031_wh**	759'969'197	2'029'774'334	1'269'805'137	476'045'828	428'999'287	47'046'541	wh	*C. ibex*
**nu.ibx20**	645'141'286	764'369'563	119'228'277	4'946'168	4'946'168	0	NA	*C. nubiana*
**nu.ibx61**	812'058'278	1'328'614'042	516'555'764	260'303'299	260'303'299	0	NA	*C. nubiana*
**py.Z5_hb**	815'478'576	1'896'275'992	1'080'797'416	493'413'944	408'045'097	85'368'847	hb	*C. pyrenaica*
**py.M518_sn**	332'469'317	2'347'523'839	2'015'054'522	845'012'933	831'829'914	13'183'019	sn	*C. pyrenaica*
**py.V53_vi**	819'670'708	1'640'855'629	821'184'921	186'497'356	186'497'356	0	vi	*C. pyrenaica*
**py.V66_vi**	736'060'943	1'618'831'409	882'770'466	256'608'832	227'041'412	29'567'420	vi	*C. pyrenaica*
**si.Casi1**	255'207'615	408'162'919	152'955'304	75'253'542	51'229'618	24'023'924	NA	*C. sibirica*
**si.Casi2**	1'006'207'772	1'244'095'194	237'887'422	38'624'317	38'624'317	0	NA	*C. sibirica*
**IRCA-C3-1001**	149'214'281	673'260'511	524'046'230	367'252'324	276'738'287	90'514'037	NA	*C. aegagrus*
**IRCA-G2-5063**	195'537'312	445'690'958	250'153'646	95'027'576	84'593'640	10'433'936	NA	*C. aegagrus*
**IRCA-I11-0001**	68'939'368	846'990'938	778'051'570	541'984'632	454'595'429	87'389'203	NA	*C. aegagrus*
**IRCA-I6-5237**	120'388'494	641'820'877	521'432'383	336'343'434	324'887'694	11'455'740	NA	*C. aegagrus*
**IRCA-K12-0005**	74'421'878	779'866'962	705'445'084	465'174'475	384'455'993	80'718'482	NA	*C. aegagrus*
**IRCA-M12-0008**	93'266'760	338'597'836	245'331'076	97'126'960	97'126'960	0	NA	*C. aegagrus*
**fal.Cafal1**	86'633'715	2'391'817'852	2'305'184'137	1'702'176'670	1'340'824'547	361'352'123	NA	*C. falconeri*
**FRCH-AL-0002**	193'289'812	416'876'710	223'586'898	150'003'969	150'003'969	0	Alpine	*C. aegagrus hircus*
**IRCH-B3-5031**	102'635'552	113'614'146	10'978'594	0	0	0	NA	*C. aegagrus hircus*
**IRCH-C5-5206**	80'643'047	824'805'115	744'162'068	614'532'865	537'104'184	77'428'681	NA	*C. aegagrus hircus*
**IRCH-F3-5044**	89'815'929	342'912'438	253'096'509	181'835'799	165'180'180	16'655'619	NA	*C. aegagrus hircus*
**IRCH-F4-5093**	71'749'818	727'932'777	656'182'959	511'594'515	395'940'769	115'653'746	NA	*C. aegagrus hircus*
**IRCH-G5-5185**	124'475'913	147'157'579	22'681'666	2'708'642	2'708'642	0	NA	*C. aegagrus hircus*
**MOCH-AA10-2195**	146'220'987	340'288'629	194'067'642	151'554'435	114'959'593	36'594'842	landrace	*C. aegagrus hircus*
**MOCH-L10-3100**	120'386'963	1'032'323'265	911'936'302	708'108'515	574'495'587	133'612'928	landrace	*C. aegagrus hircus*
**MOCH-Q15-1143**	118'049'121	1'156'701'237	1'038'652'116	878'378'405	615'022'573	263'355'832	landrace	*C. aegagrus hircus*
**MOCH-R5-0037**	115'316'268	542'454'919	427'138'651	341'618'532	278'102'708	63'515'824	landrace	*C. aegagrus hircus*
**MOCH-S8-2252**	114'340'854	1'082'372'825	968'031'971	800'852'904	593'627'379	207'225'525	landrace	*C. aegagrus hircus*
**MOCH-V5-3059**	178'078'661	256'774'033	78'695'372	21'118'017	21'118'017	0	landrace	*C. aegagrus hircus*
**hi.PCGA06**	828'868'429	990'581'676	161'713'247	91'561'239	91'561'239	0	Peacock	*C. aegagrus hircus*
**FRCH-SA-0001**	172'403'707	326'762'731	154'359'024	90'564'251	57'648'233	32'916'018	Saanen	*C. aegagrus hircus*
**ITCH-SA-0001**	198'698'577	342'352'391	143'653'814	90'281'895	78'559'944	11'721'951	Saanen	*C. aegagrus hircus*
**ITCH-SA-0005**	186'683'295	307'887'462	121'204'167	79'505'337	79'505'337	0	Saanen	*C. aegagrus hircus*

Supplementary Table 4: **Deleterious mutations segregating among and within species.** SNPs were filtered as detailed in the Methods. For Alpine and Iberian ibex, we jointly defined a subset of SNPs for which either the Alpine and/or the Iberian ibex carried the derived allele. The SNPs include only sites for which no other species than Alpine and Iberian ibex carried the derived allele. See Methods for details on ancestral/derived state identification procedures.

Variable sites within groups/species:	All species			*C. ibex*		*C. ibex* derived		*C. pyrenaica*		*C. pyrenaica* derived		*C. hircus*		*C. aegagrus*		*C. sibirica*		*C. falconeri*		*C. nubiana*	
	number of SNPs	%		number of SNPs	%	number of SNPs	%	number of SNPs	%	number of SNPs	%	number of SNPs	%	number of SNPs	%	number of SNPs	%	number of SNPs	%	number of SNPs	%
**Total**	**370'853**			**22'255**		**16'545**		**29'100**		**23'656**		**123'791**		**83'088**		**58'850**		**11'062**		**43'141**	
**SnpEff category**																					
Stop gained	444	0.12		60	0.27	38	0.23	60	0.21	39	0.16	163	0.13	108	0.13	70	0.12	29	0.26	58	0.13
High	614	0.17		69	0.31	41	0.25	72	0.25	47	0.2	241	0.19	156	0.19	102	0.17	37	0.33	76	0.18
Moderate	70'873	19.1		5157	23.2	3754	22.7	6546	22.5	5254	22.2	24540	19.8	15841	19.1	10491	17.8	2358	21.3	8457	19.6
Low	122'677	33.1		7186	32.3	5068	30.6	9236	31.7	7214	30.5	41614	33.6	28435	34.2	20332	34.5	3612	32.7	14631	33.9
Modifier	176'689	47.6		9843	44.2	7682	46.4	13246	45.5	11141	47.1	57396	46.4	38656	46.5	27925	47.5	5055	45.7	19977	46.3
**Range of GERP conservation score**																					
>6	4'343	1.2		312	1.4	218	1.3	369	1.3	293	1.2	1494	1.2	989	1.2	695	1.2	125	1.1	507	1.2
4 - 6	96'528	26.0		5759	25.9	4277	25.9	7545	25.9	6151	26.0	32303	26.1	21652	26.1	15493	26.3	2874	26.0	11148	25.8
2 - 4	96'967	26.1		6040	27.1	4598	27.8	7901	27.2	6492	27.4	32525	26.3	21437	25.8	14748	25.1	2814	25.4	11205	26.0
-2 - 2	173'015	46.7		10144	45.6	7452	45.0	13285	45.7	10720	45.3	57469	46.4	39010	47.0	27914	47.4	5249	47.5	20281	47.0

Supplementary Table 5: **Deleterious mutations (indels) segregating among and within species.** Identical to Supplementary Table 4 except showing indels instead of SNPs.

Variable sites within groups/species:	All species			*C. ibex*		*C. ibex* derived		*C. pyrenaica*		*C. pyrenaica* derived		*C. hircus*		*C. aegagrus*		*C. sibirica*		*C. falconeri*		*C. nubiana*	
	number of indels	%		number of indels	%	number of indels	%	number of indels	%	number of indels	%	number of indels	%	number of indels	%	number of indels	%	number of indels	%	number of indels	%
**Total**	**27'039**			**3'618**		**2'012**		**2'981**		**1'960**		**8'827**		**5'730**		**4'746**		**1'052**		**3'632**	
**SnpEff category**																					
Stop gained	32	0.12		4	0.11	3	0.15	2	0.07	2	0.1	9	0.1	8	0.14	11	0.23	0	0	0	0
High	1'890	6.99		651	17.99	280	13.92	354	11.88	119	6.07	825	9.35	500	8.73	394	8.3	186	17.68	375	10.32
Moderate	1'273	4.7		248	6.9	119	5.9	178	6.0	103	5.3	491	5.6	265	4.6	215	4.5	62	5.9	185	5.1
Low	95	0.4		12	0.3	5	0.2	14	0.5	11	0.6	25	0.3	17	0.3	19	0.4	3	0.3	15	0.4
Modifier	23'781	88		2'707	74.8	1608	79.9	2'435	81.7	1727	88.1	7486	84.8	4'948	86.4	4'118	86.8	801	76.1	3'057	84.2
**Range of GERP conservation score**																					
>6	15'141	56		1'723	47.6	987	49.1	1'551	52.0	1053	53.7	4835	54.8	3'246	56.6	2'706	57.0	562	53.4	2'067	56.9
4 - 6	7'358	27.2		975	26.9	534	26.5	821	27.5	546	27.9	2427	27.5	1524	26.6	1'259	26.5	268	25.5	933	25.7
2 - 4	4'357	16.1		877	24.2	470	23.4	587	19.7	346	17.7	1508	17.1	917	16.0	750	15.8	212	20.2	608	16.7
-2 - 2	183	0.7		43	1.2	21	1.0	22	0.7	15	0.8	57	0.6	43	0.8	31	0.7	10	1.0	24	0.7

Supplementary Table 6: **Predicted protein functions.** Predicted protein functions of genes for which at least one homozygote was found for the derived, high-impact allele.

Gene name	Predicted protein function	Polymorphism within species	SnpEff stop-gained
**Alpine ibex (*C. ibex*)**			
**KNG1**	kininogen-1 isoforms X1-3	Segregating	yes
**LOC102181723**	protein FAM188B2	Fixed	yes
**LOC102181167**	60S ribosomal protein L10a-like	Fixed	yes
**DIS3L2**	DIS3-like exonuclease 2	Segregating	yes
**EPHA8**	ephrin type-A receptor 8	Segregating	yes
**LOC102178786**	coiled-coil domain-containing protein 30-like	Segregating	no
**LOC102180106**	unknown	Segregating	no
**OVCH1**	ovochymase-1	Fixed	yes
**ERAP2**	endoplasmic reticulum aminopeptidase 2	Segregating	no
**TLN2**	talin-2	Segregating	yes
**POLR1A**	DNA-directed RNA polymerase I subunit RPA1	Segregating	yes
**LOC102188952**	unknown	Segregating	yes
**OOEP**	oocyte-expressed protein homolog	Segregating	yes
**FOXR1**	Forkhead box protein R1	Segregating	yes
**PNMAL1**	PNMA-like protein 1	Segregating	yes
**RAD1**	cell cycle checkpoint protein RAD1	Fixed	no
**KIF9**	kinesin-like protein KIF9 isoforms X1-3	Fixed	yes
**LOC102179713**	C-C chemokine receptor type 1-like	Segregating	yes
**CD2BP2**	CD2 antigen cytoplasmic tail-binding protein 2 isoforms X1-2	Fixed	yes
**LOC102189088**	zinc finger protein 205 isoform X1	Fixed	no
**LOC102188472**	synaptotagmin-15 isoforms X1-4	Segregating	yes
**LOC102175191**	solute carrier family 22 member 9-like	Fixed	yes
**Iberian Ibex (*C. pyrenaica*)**			
**LOC102181723**	protein FAM188B2	Segregating	yes
**LOC102181167**	60S ribosomal protein L10a-like	Segregating	yes
**CAPZB**	F-actin-capping protein subunit beta	Segregating	yes
**TMBIM6**	bax inhibitor 1	Segregating	yes
**LOC102191021**	protein HP-25 homolog 2	Segregating	yes
**OVCH1**	ovochymase-1	Fixed	yes
**LOC102173270**	C-type lectin domain family 2 member B	Segregating	yes
**TLR6**	toll-like receptor 6	Segregating	yes
**TLR6**	toll-like receptor 6 isoform X1	Segregating	yes
**LOC106502316**	zinc finger protein 77-like	Segregating	yes
**LOC102191525**	eukaryotic translation initiation factor 3 subunit J	Segregating	yes
**TMEM244**	transmembrane protein 244	Segregating	yes

Gene name	Predicted protein function	Polymorphism within species	SnpEff stop-gained
**IGF2R**	cation-independent mannose-6-phosphate receptor	Fixed	yes
**PPP1R14D**	protein phosphatase 1 regulatory subunit 14D	Segregating	yes
**LOC102187409**	60S ribosomal protein L36a-like	Segregating	yes
**LOC102173760**	Fer-1-like protein 4	Segregating	yes
**SOGA1**	protein SOGA1	Segregating	yes
**CCDC73**	coiled-coil domain-containing protein 73	Segregating	yes
**PER3**	period circadian protein homolog 3	Segregating	yes
**LOC102178853**	arachidonate 12-lipoxygenase epidermal-type-like	Segregating	yes
**LOC102171368**	keratin-associated protein 4-9-like	Fixed	yes
**LOC102177379**	myeloid-associated differentiation marker-like	Segregating	yes
**KIF9**	kinesin-like protein KIF9 isoforms X1-3	Fixed	yes
**CLN3**	battenin isoforms X1-3	Segregating	yes
**CD2BP2**	CD2 antigen cytoplasmic tail-binding protein 2 isoforms X1-2	Fixed	yes
**LOC102189088**	zinc finger protein 205 isoform X1	Segregating	no
**LOC102170020**	deleted in malignant brain tumors 1 protein-like	Segregating	no

Supplementary Note 1: Script used for individual based simulations.

```
run_mode overwrite
random_seed 30317
logfile logfile.log
root_dir SIMS/sim5000_0.0014_0.01
filename sim


replicates 100
generations 3023
patch_number 12
patch_capacity (@g0 {{1000, 0, 0, 0, 0, 0, 0, 0, 0, 0, 0,0}}, @g3000 {{500, 0, 0, 0, 0, 0, 0, 0, 0, 0, 0,0}}, @g3005 {{80,
    0, 0, 0, 0, 0, 0, 0, 0, 0, 0,0}}, @g3007 {{1000, 0, 0, 0, 0, 0, 0, 0, 0, 0, 0,0}}, @g3010 {{1000, 25, 30, 0, 0, 0, 0, 0, 0,
    0, 20,0}}, @g3012 {{1000, 25, 30, 85, 73, 46, 0, 0, 0, 0, 20,0}}, @g3017 {{1000, 25, 30, 85, 73, 46, 41, 30, 22,
    116, 20, 43}})


ntrl_init 1
breed_selection 2
disperse 3
aging 4
save_stats 5
save_files 6

mating_system 1
mean_fecundity 5
dispersal_connectivity_matrix  (@g0 {{1}{2}{3}{4}{5}{6}{7}{8}{9}{10}{11}{12}},@g3010
    {{1,2,3,11}{2}{3}{4}{5}{6}{7}{8}{9}{10}{11}{12}},@g3011
    {{1}{2}{3}{4}{5}{6}{7}{8}{9}{10}{11}{12}},@g3012
    {{1}{2,6,4,5}{3,6,4,5}{4}{5}{6}{7}{8}{9}{10}{11}{12}},@g3013
    {{1}{2}{3}{4}{5}{6}{7}{8}{9}{10}{11}{12}},@g3017
    {{1,5}{2}{3}{4,7}{5,8}{6,9,12,10}{7}{8}{9}{10}{11}{12}},@g3018
    {{1}{2}{3}{4}{5}{6}{7}{8}{9}{10}{11}{12,9}},@g3019 {{1}{2}{3}{4}{5}{6}{7}{8}{9}{10}{11}{12}})
dispersal_reduced_matrix  (@g0 {{1}{1}{1}{1}{1}{1}{1}{1}{1}{1}{1}{1}},@g3010
    {{0.854,0.060,0.076,0.010}{1}{1}{1}{1}{1}{1}{1}{1}{1}{1}{1}},@g3011
    {{1}{1}{1}{1}{1}{1}{1}{1}{1}{1}{1}{1}},@g3012
    {{1}{0.035,0.400,0.353,0.212}{0.333,0.49,0.059,0.118}{1}{1}{1}{1}{1}{1}{1}{1}{1}},@g3013
    {{1}{1}{1}{1}{1}{1}{1}{1}{1}{1}{1}{1}},@g3017
    {{0.996,0.004}{1}{1}{0.910,0.090}{0.839,0.161}{0.361,0.051,0.217,0.371}{1}{1}{1}{1}{1}{1}},@g3018
    {{1}{1}{1}{1}{1}{1}{1}{1}{1}{1}{1}{0.754,0.246}},@g3019 {{1}{1}{1}{1}{1}{1}{1}{1}{1}{1}{1}{1}})

## SELECTION TRAITS
selection_trait delet
selection_model direct
selection_fitness_model absolute


## NEUTRAL TRAITS
ntrl_loci 500
ntrl_all 2
ntrl_mutation_rate 5e-05
ntrl_recombination_rate 0.5
ntrl_mutation_model 1
ntrl_init_patch_freq {{0.0014}}

## DELETERIOUS TRAITS
delet_loci 5000
delet_mutation_rate 5e-05
delet_backmutation_rate 5e-07 # default
delet_recombination_rate 0.5
```

delet_mutation_model 1
delet_fitness_model 1

delet_init_freq 0.0014
delet_effects_distribution gamma
delet_effects_mean 0.01
delet_dominance_mean 0.37

delet_effects_dist_param1 0.3

OUTPUT
stat_dir stats
stat_log_time (@g0 1000, @g3000 1)
stat adlt.demography pop.patch fecundity migrants.patch migrants adlt.delet viability ntrl.freq
stat_output_compact
stat_output_CSV

ntrl_save_genotype FSTAT
ntrl_output_dir ntrl_geno
ntrl_output_logtime {{1, 1000, 2000, 2999, 3000, 3001, 3002, 3003, 3004, 3005, 3006, 3007, 3008, 3009, 3010, 3011, 3012, 3013, 3014, 3015, 3016, 3017, 3018, 3019, 3020, 3021, 3022, 3023}}

delet_save_genotype
delet_genot_dir delet_geno
delet_genot_logtime {{1, 1000, 2000, 2999, 3000, 3001, 3002, 3003, 3004, 3005, 3006, 3007, 3008, 3009, 3010, 3011, 3012, 3013, 3014, 3015, 3016, 3017, 3018, 3019, 3020, 3021, 3022, 3023}}

Supplementary References

1. Piodi, M., 1984. Le Bouquetin des Alpes - *Capra ibex*. In: C.I.C. Kommission Grosswild (Editor), Der Steinbock in Eurasien. C.I.C. Symposium, Pontresina, Switzerland, 24-25 February 1984
2. Stuwe, M. & Nievergelt, B. Recovery of alpine ibex from near extinction - the result of effective protection, captive breeding, and reintroductions. Applied Animal Behaviour Science **29,** 379–387 (1991).
3. Aeschbacher, S., Futschik, A. & Beaumont, M. A. Approximate Bayesian computation for modular inference problems with many parameters: the example of migration rates. Mol Ecol **22,** 987–1002 (2013). DRYAD entry doi:10.5061/dryad.274b1
4. Terrier, G. & Rossi, P. Le bouquetin (*Capra ibex ibex*) dans les alpes maritimes franco-italiennes: occupation de l'éspace, clonisation et régulation naturelles. Traveaux Scientifiques du Parc National de la Vanoise **XVIII,** 271–288 (1994).
5. Alpi Marittime Natural Park
6. Biebach, I. & Keller, L. F. Inbreeding in reintroduced populations: the effects of early reintroduction history and contemporary processes. Conserv Genet **11,** 527–538 (2010).
7. Couturier M. Le bouquetin des Alpes. Edited by author, Grenoble, France, (1962).
8. Pérez JM, Granados JE, Soriguer RC, Fandos P, Márquez FJ, Crampe JP, et al. Distribution, status and conservation problems of the Spanish Ibex, *Capra pyrenaica* (Mammalia: Artiodactyla). Mammal Review **32,** 26–39 (2002);.
9. Peña, J. *La cabra montés en España.* International Game Congress, Switzerland (1978).
10. Ortuño, F. & Peña, J. Reservas y cotos nacionales de caza. INCAFO, Madrid, UK. (1979)
11. Alados CL. Distribution and status of the Spanish ibex (*Capra pyrenaica*, Schinz). In: The Biology and Management of Mountain Ungulates (Ed. by Lovari S.), pp. 204–211. Croom-Helm, Beckenham London, UK, (1985).
