# Supplementary material for: Purging of highly deleterious mutations through severe bottlenecks in Alpine ibex
Source: Nat Commun. 2020 Feb 21;11:1001. doi: 10.1038/s41467-020-14803-1 (PMC7035315; doi:10.1038/s41467-020-14803-1)
Supplement: Supplementary file 3 — Reporting Summary [file 41467_2020_14803_MOESM3_ESM.pdf]

nature research

Corresponding author(s): Christine Grossen, Daniel Croll

Last updated by author(s): Dec 13, 2019

Reporting Summary

Nature Research wishes to improve the reproducibility of the work that we publish. This form provides structure for consistency and transparency in reporting. For further information on Nature Research policies, see Authors & Referees and the Editorial Policy Checklist .

Statistics

For all statistical analyses, confirm that the following items are present in the figure legend, table legend, main text, or Methods section.

n/a	Confirmed	
☐	☒	The exact sample size (*n*) for each experimental group/condition, given as a discrete number and unit of measurement
☐	☒	A statement on whether measurements were taken from distinct samples or whether the same sample was measured repeatedly
☐	☒	The statistical test(s) used AND whether they are one- or two-sided<br>*Only common tests should be described solely by name; describe more complex techniques in the Methods section.*
☒	☐	A description of all covariates tested
☐	☒	A description of any assumptions or corrections, such as tests of normality and adjustment for multiple comparisons
☐	☒	A full description of the statistical parameters including central tendency (e.g. means) or other basic estimates (e.g. regression coefficient) AND variation (e.g. standard deviation) or associated estimates of uncertainty (e.g. confidence intervals)
☐	☒	For null hypothesis testing, the test statistic (e.g. *F*, *t*, *r*) with confidence intervals, effect sizes, degrees of freedom and *P* value noted<br>*Give P values as exact values whenever suitable.*
☒	☐	For Bayesian analysis, information on the choice of priors and Markov chain Monte Carlo settings
☒	☐	For hierarchical and complex designs, identification of the appropriate level for tests and full reporting of outcomes
☐	☒	Estimates of effect sizes (e.g. Cohen's *d*, Pearson's *r*), indicating how they were calculated

Our web collection on statistics for biologists contains articles on many of the points above.

Software and code

Policy information about availability of computer code

Data collection	Simulated datasets were generated using NEMO v. 2.3.5.1
Data analysis	Raw reads were trimmed using Trimmomatic v. 0.36<br>Reads were aligned using bowtie v. 2.2.5<br>Duplicate reads were marked using Picard-tools v. 1.130<br>SNP calling was performed using GATK v. 3.6 and Freebayes v 1.0.2-33-gdbb6160<br>Runs of homozygosity were identified using bcftools v. 1.3.1<br>SNP effects were determined using snpEff v. 4.3<br>Protein homology was determined using InterProScan v. 5.33<br>GERP score annotations were transferred using liftOver picard-tools-1.130<br>RNAseq data was mapped using hisat v. 2.0.5<br>Read counts were generated using subread v. 1.5.1<br>Read normalization was performed using the R package edgeR<br>Parameter space for simulations was explored using aNEMOne

For manuscripts utilizing custom algorithms or software that are central to the research but not yet described in published literature, software must be made available to editors/reviewers. We strongly encourage code deposition in a community repository (e.g. GitHub). See the Nature Research guidelines for submitting code & software for further information.

Data

Policy information about availability of data

All manuscripts must include a data availability statement. This statement should provide the following information, where applicable:

- Accession codes, unique identifiers, or web links for publicly available datasets
- A list of figures that have associated raw data
- A description of any restrictions on data availability

Raw whole-genome sequencing data produced for this project was deposited at the NCBI Short Read Archive under the Accession nos. SAMN10736122–SAMN10736160 (BioProject PRJNA514886).
Raw RNA sequencing data produced for this project was deposited at the NCBI Short Read Archive under the Accession nos. SAMN10839218-SAMN10839227 (BioProject PRJNA517635).

Field-specific reporting

Please select the one below that is the best fit for your research. If you are not sure, read the appropriate sections before making your selection.

☐ Life sciences ☐ Behavioural & social sciences ☒ Ecological, evolutionary & environmental sciences

For a reference copy of the document with all sections, see nature.com/documents/nr-reporting-summary-flat.pdf

Ecological, evolutionary & environmental sciences study design

All studies must disclose on these points even when the disclosure is negative.

Study description	Whole-genome sequencing of ibex, wild and domestic goats was used to analyze deleterious mutations segregating within species.
Research sample	Capra ibex: N=29, C. pyrenaica: N=4, C. aegagrus: N=6, C. sibirica: N=2, C. falconeri: N=1, C. nubiana: N=2, C. hircus N=16
Sampling strategy	Sample sizes (see above) reflect our focus. Our study was primarily based on C. ibex, hence the dominance in the sampling. For each species, individuals were included that overall covered the geographic range of the species. For domestic goats, a set of individuals representing the known genetic diversity was chosen.
Data collection	The following whole-genome sequencing was performed in the frame of our study: Capra ibex: N=29, C. pyrenaica: N=4, C. aegagrus: N=6, C. sibirica: N=2, C. falconeri: N=1, C. nubiana: N=2.<br>Whole-genome sequencing of domestic goats (C. hircus) was performed by the NextGen Consortium (https://nextgen.epfl.ch). The corresponding raw data was downloaded from the EBI Short Read Archive: ftp://ftp.sra.ebi.ac.uk/vol1/fastq/
Timing and spatial scale	Ibex and wild goat samples were collected between 2003-2015.
Data exclusions	No data was excluded.
Reproducibility	No experiments were performed. A simulation study was performed to ascertain whether the empirical findings can be reproduced with a realistically parametrized model.
Randomization	Individuals were grouped into their population of origin and species. No randomization was performed.
Blinding	Not applicable.

Did the study involve field work? ☐ Yes ☒ No

Reporting for specific materials, systems and methods

We require information from authors about some types of materials, experimental systems and methods used in many studies. Here, indicate whether each material, system or method listed is relevant to your study. If you are not sure if a list item applies to your research, read the appropriate section before selecting a response.

Materials & experimental systems

n/a	Involved in the study
☒	☐ Antibodies
☒	☐ Eukaryotic cell lines
☒	☐ Palaeontology
☒	☐ Animals and other organisms
☒	☐ Human research participants
☒	☐ Clinical data

Methods

n/a	Involved in the study
☒	☐ ChIP-seq
☒	☐ Flow cytometry
☒	☐ MRI-based neuroimaging
